# SnapFISH: a computational pipeline to identify chromatin loops from multiplexed DNA FISH data

**DOI:** 10.1038/s41467-023-40658-3

**Published:** 2023-08-12

**Authors:** Lindsay Lee, Hongyu Yu, Bojing Blair Jia, Adam Jussila, Chenxu Zhu, Jiawen Chen, Liangqi Xie, Antonina Hafner, Shreya Mishra, Duan Dennis Wang, Caterina Strambio-De-Castillia, Alistair Boettiger, Bing Ren, Yun Li, Ming Hu

**Affiliations:** 1 Department of Quantitative Health Sciences, Lerner Research Institute, Cleveland Clinic Foundation, Cleveland, OH USA; 2 Department of Statistics, University of Wisconsin Madison, Madison, WI USA; 3 Department of Biochemistry, University of Wisconsin Madison, Madison, WI USA; 4 Bioinformatics and Systems Biology Graduate Program, University of California San Diego, La Jolla, CA USA; 5 Medical Scientist Training Program, University of California San Diego, La Jolla, CA USA; 6 Ludwig Institute for Cancer Research, La Jolla, CA USA; 7 New York Genome Center, New York, NY USA; 8 Department of Physiology and Biophysics, Institute for Computational Biomedicine, Weill Cornell Medicine, New York, NY USA; 9 Department of Biostatistics, University of North Carolina, Chapel Hill, NC USA; 10 Department of Infection Biology, Lerner Research Institute, Cleveland Clinic Foundation, Cleveland, OH USA; 11 Department of Cancer Biology, Lerner Research Institute, Cleveland Clinic Foundation, Cleveland, OH USA; 12 Department of Developmental Biology, Stanford University, Stanford, CA USA; 13 Chapel Hill High School, Chapel Hill, NC USA; 14 Program in Molecular Medicine, University of Massachusetts Chan Medical School, Worcester, MA USA; 15 Center for Epigenomics & Department of Cellular and Molecular Medicine, University of California San Diego, La Jolla, CA USA; 16 Department of Genetics, University of North Carolina, Chapel Hill, NC USA; 17 Department of Computer Science, University of North Carolina, Chapel Hill, NC USA

**Keywords:** Software, Statistical methods, Bioinformatics, Epigenomics, Genetic techniques

## Abstract

Multiplexed DNA fluorescence in situ hybridization (FISH) imaging technologies have been developed to map the folding of chromatin fibers at tens of nanometers and up to several kilobases in resolution in single cells. However, computational methods to reliably identify chromatin loops from such imaging datasets are still lacking. Here we present a Single-Nucleus Analysis Pipeline for multiplexed DNA FISH (SnapFISH), to process the multiplexed DNA FISH data and identify chromatin loops. SnapFISH can identify known chromatin loops from mouse embryonic stem cells with high sensitivity and accuracy. In addition, SnapFISH obtains comparable results of chromatin loops across datasets generated from diverse imaging technologies. SnapFISH is freely available at https://github.com/HuMingLab/SnapFISH.

## Abstract (web summary)

Multiplexed DNA FISH technologies are powerful tools to reveal chromatin spatial organisation. Here, the authors developed SnapFISH, a computational pipeline to identify chromatin loops from multiplexed DNA FISH data.

## Introduction

How chromatin folds inside the nucleus is a fundamental question in the study of genome structure and function^1^. Disruption of chromatin organization can lead to gene dysregulation, and has been associated with a variety of human developmental disorders, neuropsychiatric diseases, and cancers^2^. Different from proximity-ligation assays^3,4^ that infer the 3D genome through *indirect* measurement of DNA sequence contacts, chromatin tracing, as an emerging microscopy-based technology, can visualize bright spots corresponding to individual targeted genomic segments arrayed along chromatin fibers, and map their physical location in three-dimensional space. This rich imaging data permits the *direct* measurement of Euclidean distances between targeted genomic segments of interest—such as promoters and distal *cis*-regulatory elements - allowing an intimate look into the organization of chromosomes^5^. During the last decade, a number of chromatin tracing technologies have emerged, including multiplexed DNA fluorescence in situ hybridization (FISH)^6,7^, DNA-MERFISH^8^, DNA seqFISH+^9,10^, ORCA^11^, MINA^12^, Hi-M^13^, OligoFISSEQ^14^ and IGS^15^ (more details can be found in recent review articles^5,16,17^). These techniques can resolve the spatial location of discrete targeted genomic segments with tens of nanometer precision in single cells. They have been used to image the entire mammalian genome at megabase (Mb) resolution^9,10^, one full chromosome at 50 kilobase (Kb) resolution^8^, and a few selected regions at 2.5 Kb ~ 30 Kb resolution^7–11,18,19^, promising to uncover novel insights into chromatin folding and its role in gene regulation^5^.

Chromatin loops are a key structural feature of chromatin spatial organization, and may serve as the structural basis of gene regulation. Originally discovered from bulk Hi-C data^4,20^ as “dots” at the corners of topologically associating domains, and recently identified from single cell Hi-C data^21,22^ and imaging data^18^, chromatin loops are defined as pairs of genomic loci with closer spatial proximity compared to other pairs of loci in the local neighborhood region^4,20^. Chromatin loops between enhancers and promoters have been used to infer target genes for distal enhancers^23^. Chromatin loops between CTCF binding sites are associated with the formation of topological associating domains^24^, which are megabase sized chromatin domains constraining enhancer-promoter interactions. Extensive studies have demonstrated that chromatin loops play an essential role in maintaining the 3D structure of the genome and facilitating gene regulation^25–28^.

The functional importance of chromatin loops makes it important to develop loop callers tailored to different input experimental datasets (Supplementary Information Section 1). All existing loop callers are designed for genomic data generated from proximity-ligation assays, which utilize the *count*-based statistical framework to model chromatin contact frequency^29^. As a result, these tools are inherently not directly applicable to imaging data, which allow the *continuous* measurement of Euclidean distances between targeted genomic segments of interest. To the best of our knowledge, no method is available to identify chromatin loops from such imaging data. In the wake of the rapid development of chromatin tracing technologies, tailored computational methods to reliably identify chromatin loops from imaging data have become more critical. Importantly, such loop analysis methods may advance our understanding of the relationships between genome structure and gene regulation.

In this work, to fill in the abovementioned methodological gap, we develop Single-Nucleus Analysis Pipeline for multiplexed DNA FISH data (SnapFISH), a computational pipeline to identify chromatin loops from multiplexed DNA FISH data. SnapFISH can identify known chromatin loops from mouse embryonic stem cells with high sensitivity and accuracy. In addition, SnapFISH can accommodate datasets generated from diverse imaging technologies.

## Results

### SnapFISH algorithm

**Fig. 1:**
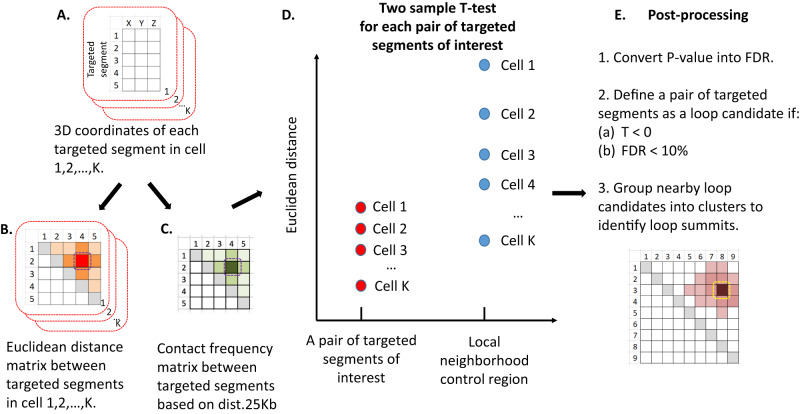
The flowchart of the SnapFISH algorithm. **A** The 3D coordinates for each targeted segment in cell 1, 2, …, *K*. Each matrix represents one cell. Each row is one targeted segment and *X*, *Y*, *Z* are the 3D coordinates. **B** Euclidean distance matrices. Again, each matrix is for one cell. The dashed purple block highlighted the pair of targeted segments of interest: between the targeted segment 2 and the targeted segment 4. **C** Population-level contact frequency matrix. Similar to **B**, the dashed purple block highlighted the pair of targeted segments of interest: between the targeted segment 2 and the targeted segment 4. dist.25 Kb: the average Euclidean distance between two targeted segments with the 1D genomic distance of 25 Kb. **D** Two-sample *T*-test comparing the Euclidean distance between the pair of targeted segments of interest and its local neighborhood control region. **E** Post-processing to identify loop candidates and loop summits. The cartoon represents the clustering of nearby loop candidates and the selection of loop summit (i.e., between the targeted segment 3 and the targeted segment 8), which is highlighted by the dashed yellow block (see detailed listed in Methods). Source data are provided as a Source Data file.

In the same spirit of our recently developed SnapHiC^21^ pipeline for single cell Hi-C data, SnapFISH also treats each imaged cell as an independent unit, to boost the statistical power of identifying chromatin loops. Specifically, SnapFISH takes multiplexed DNA FISH single bright spot localization data as input, and outputs the predicted chromosomal location of chromatin loops. Briefly, SnapFISH first collects the 3D localization coordinates of each genomic segment targeted by FISH (hereafter referred to as targeted segment) in each cell (Fig. 1A), and computes the pairwise Euclidean distances between all imaged targeted segments (Fig. 1B). SnapFISH then calculates the average Euclidean distance between two targeted segments with the 1D genomic distance of 25Kb (termed as avg.dist.1D.25Kb), and defines the population-level contact frequency between any two targeted segments as the fraction of cells with Euclidean distance smaller than avg.dist.1D.25Kb (Fig. 1C) (see details in a recent preprint^30^). Next, for all pairs of targeted segments found within a 1D genomic distance range of 100 Kb ~ 1 Mb, SnapFISH compares the pairwise Euclidean distances between the pair of interest and its local neighborhood region (Supplementary Fig. S1, see details in Methods) using a two-sample *T*-test (Fig. 1D). SnapFISH then converts the resulting P-values into false discovery rates (FDRs), and defines a pair of targeted segments as a loop candidate if the average Euclidean distance between the pair of interests is smaller than the average Euclidean distance in its local neighborhood region (i.e., *T*-test statistic <0), and FDR is less than 10% (Fig. 1E, see details in Methods). Lastly, SnapFISH groups nearby loop candidates into clusters, identifies the pair with the lowest FDR within each cluster (hereafter referred to as the cluster summit), and uses both cluster summits and singletons (i.e., cluster with only one loop candidate) as the final list of chromatin loops (Fig. 1E, see details in Methods).

### Optimizing the SnapFISH algorithm using DNA seqFISH+ data in mESCs

**Fig. 2:**
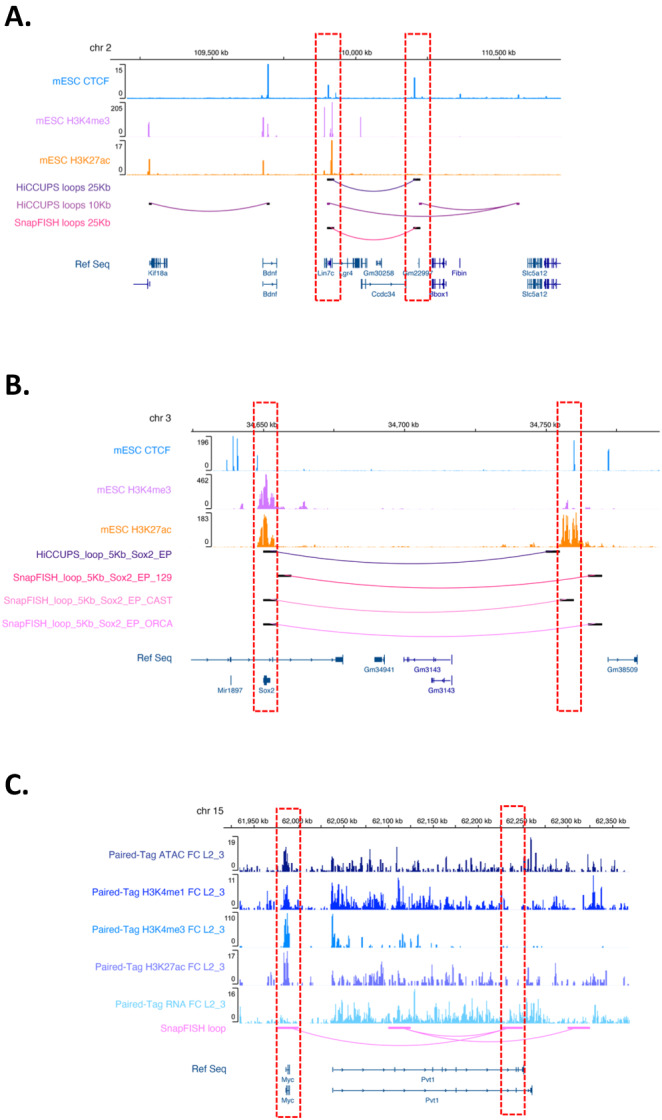
SnapFISH identified chromatin loops with high accuracy. **A** SnapFISH identified a CTCF-CTCF loop in chromosome 2 from mESC DNA seqFISH+ data. The top three tracks are mESC CTCF, mESC H3K4me3 and mESC H3K27ac ChIP-seq data. The middle three tracks represent HiCCUPS-identified loops at 25 Kb bin resolution, HiCCUPS-identified loops at 10 Kb bin resolution, and SnapFISH-identified loops at 25 Kb bin resolution, respectively. The bottom track is the Refseq gene annotation. Both anchors (dashed red boxes) contain mESC CTCF ChIP-seq peaks. Source data are provided as a Source Data file. **B** SnapFISH identified the *Sox2* enhancer-promoter loop from mESC multiplexed DNA FISH data and mESC ORCA data. The top three tracks are mESC CTCF, mESC H3K4me3, and mESC H3K27ac ChIP-seq data. The middle four tracks represent the HiCCUPS-identified loop from mESC bulk Hi-C data^31^, the SnapFISH-identified loop from 1,416 129 alleles and 1416 CAST alleles, and the SnapFISH-identified loop from mESC ORCA data, respectively. All these loops are at 5 Kb bin resolution. The bottom track is the Refseq gene annotation. The red dashed box on the left and the right represents the location of the promoter of the *Sox2* gene and its super-enhancer, respectively. We allow for a 5 Kb gap between loop anchors and the *Sox2* promoter or super-enhancer (see details in Methods). Source data are provided as a Source Data file. **C** SnapFISH identified an enhancer-promoter loop in chromosome 15 from DNA seqFISH+ data in mouse excitatory neurons. The top five tracks are ATAC-seq, H3K4me1, H3K4me3, H3K27ac and RNA-seq data from cortical excitatory neurons L2/3 collected from mouse frontal cortex tissue (FC_L2_3 for short)^35^. The bottom two tracks are SnapFISH-identified 25 Kb bin resolution loops and the Refseq gene annotation. The left anchor (dashed red box on the left) is at gene *Myc*. The right anchor (dashed red box on the right) is at the transcription end site of gene *Pvt1*, which contains an ATAC peak. Source data are provided as a Source Data file.

In order to optimize the SnapFISH algorithm, we re-analyzed a publicly available DNA seqFISH+ dataset from mouse embryonic stem cells (mESCs)^9^, where the authors selected one region from each chromosome, with region length ranging from 1.5 Mb ~ 2.35 Mb (Supplementary Table S1), and performed DNA seqFISH+ experiment at 25 Kb bin resolution, in two biological replicates (see details in imaging data resource). We combined data across replicates, resulting in 446 cells (i.e., 892 alleles) in total. The average targeted segment detection efficiency, defined as the proportion of imaged targeted segments among all targeted segments, is 65.2%. As the reference loop set (serving as the working truth), we re-analyzed a deeply sequenced mESC bulk Hi-C data^31^, and identified 35 loops with HiCCUPS at both 10 Kb and 25 Kb resolution in the corresponding genomic region where DNA seqFISH+ data is available (Supplementary Table S2). We applied SnapFISH to call loops from DNA seqFISH+ data in mESCs, and calculated precision, recall and F1-score based on the reference HiCCUPS loops. Among all tested combinations of SnapFISH parameters, we selected the one corresponding to the highest F1-score, defined as the harmonic mean of the precision and recall (see details in Supplementary Information Section 2 and Table S3). With the optimized parameters, SnapFISH identified 16 loops (Supplementary Table S4A), where 14 loops overlap HiCCUPS loops (Fig. 2A and Supplementary Fig. S2A**~** S2H and Fig. S3). The precision, recall and F1-score are 87.5%, 40.0% and 0.549, respectively. Specifically, 9 SnapFISH loops overlap with 16 25Kb HiCCUPS loops (recall = 56.3%), and 5 SnapFISH loops overlap with 19 10 Kb HiCCUPS loops (recall = 26.3%). As we expected, SnapFISH loops identified from imaging data from 446 cells achieved lower sensitivity compared to HiCCUPS loops identified from deeply sequenced bulk Hi-C data, which usually contains ~10^6^ cells^31^. SnapFISH achieved higher sensitivity when imaging data and bulk Hi-C data are at the same 25 Kb resolution, and most false negatives are from the finer 10 Kb resolution. In addition, we evaluated how loop strength, measured by population-level contact frequency, affects the sensitivity of SnapFISH. Supplementary Fig. S2I shows that the 14 true positives (HiCCUPS loops identified by SnapFISH) have significantly higher average population-level contact frequency (49.7%) than that (37.2%) in the 21 false negatives (HiCCUPS loops missed by SnapFISH) (two-sided two sample *T*-test *P* = 0.0014), suggesting that SnapFISH can achieve higher sensitivity for loops with higher strength.

As one illustrative example in chromosome 2, Fig. 2A shows that SnapFISH identified a CTCF-CTCF loop, which has also been detected by HiCCUPS from mESC bulk Hi-C data at 25 Kb resolution. The 3 false negatives are all 10 Kb HiCCUPS loops (Fig. 2A). In another illustrative example in chromosome 3, Supplementary Fig. S3A shows that SnapFISH identified 6 loops, where 4 loops overlap with 4 HiCCUPS loops. Notably, the 2 false positives (i.e., not overlapping HiCCUPS loops) are the CTCF-CTCF loop and enhancer-promoter loop, respectively, and both overlap chromatin interactions identified from mESC H3K4me3 PLAC-seq data^32^. Taken together, our results show that when applying to mESC DNA seqFISH+ data, SnapFISH can accurately and reliably identify loops that were previously identified from mESC bulk Hi-C data.

### Applying SnapFISH to multiplexed DNA FISH data and ORCA data in mESCs at the *Sox2* locus

Encouraged by the results from 25 Kb resolution mESC DNA seqFISH+ data, we then applied SnapFISH to the finer resolution (5 Kb) multiplexed DNA FISH data. Specifically, a multiplexed DNA FISH dataset has previously been generated from mESCs to investigate the chromatin conformation at the *Sox2* locus^18^ (such data is downloaded from the 4D Nucleome data portal, see details in imaging data resource). The 205 Kb chromosomal target region (mm10: chr3:34,601,078-34,806,078) imaged in the experiments spans both the promoter of the *Sox2* gene and its super-enhancer, which is located ~100 Kb downstream. Previous studies have identified a chromatin loop between the *Sox2* promoter and its super-enhancer via 5C^33^, and validated the functional importance of such loop via the CRISPR experiment, which showed that deletion of the super-enhancer reduces >90% of the *Sox2* expression^34^. The mESCs used in the original study^18^ have two haplotypes: the CAST allele and the 129 allele. Distinct from the abovementioned mESC DNA seqFISH+ data where one cannot differentiate between the paternal and maternal alleles, the CAST allele contains a 7.5Kb insertion containing 4 CTCF-binding sites (hereafter referred to as 4CBS) between the *Sox2* promoter and its super-enhancer, while the 129 allele does not contain the insertion. The multiplexed DNA FISH experiments involved 41 probe sets, each corresponding to individual 5 Kb genomic target segments tiling the 205 Kb region of interest. In addition, there was one extra probe for the 7.5 Kb insertion on the CAST allele, which permits the differential identification of the CAST allele and the 129 allele in the same nucleus. A previous study^18^ showed that the CAST allele-specific 7.5 Kb 4CBS insertion resulted in weaker chromatin looping strength between the *Sox2* promoter and its super-enhancer.

We re-analyzed the multiplexed DNA FISH data (see details in Methods) from a total of 1416 cells (i.e., 1416 CAST alleles and 1416 129 alleles) using SnapFISH. The average targeted segment detection efficiency is 71.9% and 69.3% for the CAST allele and the 129 allele, respectively. We first computed the average Euclidean distances (Supplementary Fig. S4A) and population-level contact frequency (Supplementary Fig. S4B) between all pairs of 5 Kb genomic targeted segment in these experiments, and compared that with the 5 Kb bin resolution chromatin contact frequency in the mESC bulk Hi-C data^18^ in an allele-specific manner (Supplementary Fig. S4C). As we expected, both average spatial distance and population-level contact frequency measured from multiplexed DNA FISH data are closely correlated with the Hi-C contact frequency. The Pearson correlation coefficient between Hi-C contact frequency and the inverse of average Euclidean distances is 0.812 and 0.805 for the CAST allele and the 129 allele, respectively. Consistently, the Pearson correlation coefficient between Hi-C contact frequency and population-level contact frequency measured from multiplexed DNA FISH data is 0.788 and 0.768 for the CAST allele and the 129 allele, respectively. Applying SnapFISH to 1,416 CAST alleles and 1,416 129 alleles identified 44 and 61 loop candidates, respectively (Supplementary Fig. S4D). Finally, SnapFISH grouped neighboring loop candidates, and detected a single chromatin loop summit between the *Sox2* promoter and the super-enhancer in both CAST and 129 alleles (Fig. 2B). As a comparison, we applied HiCCUPS to identify 5Kb bin resolution chromatin loops from mESC bulk Hi-C data^31^, and found that SnapFISH-identified loops are also near the HiCCUPS loop (Fig. 2B).

Next, we evaluated the sensitivity of SnapFISH using different numbers of input target alleles. Specifically, we ranked all 1416 CAST alleles by their targeted segment detection efficiency, and selected the top 1400, 1200, 1000, 800, 600, 400, 200, 100, and 50 CAST alleles as input for SnapFISH. We performed the same analysis with the 129 allele. Consistent with previous findings^18^ indicating that the strength of the *Sox2* enhancer-promoter loop is weaker in the CAST allele compared to that in the 129 allele, SnapFISH found fewer loop candidates among the CAST allele than that in the 129 allele (Supplementary Table S5), due to the CAST-specific 4CBS insertion. However, despite these differences, we observed that SnapFISH can accurately identify the *Sox2* enhancer-promoter loop with as few as 200 CAST alleles (Supplementary Fig. S5A) and 100 129 alleles (Supplementary Fig. S5B). Our results suggest that SnapFISH achieves high sensitivity even with a small number of cells.

Since not all targeted segments can be detected in the multiplexed DNA FISH data, we further evaluated how targeted segment detection efficiency affects the sensitivity of SnapFISH. Specifically, we again ranked all 1416 CAST alleles by their targeted segment detection efficiency, and then equally divided them into three groups, where each group consisted of 472 CAST alleles. We stratified them as “high”, “median” and “low” targeted segment detection efficiency groups, with an average targeted segment detection efficiency of 85.4%, 73.2%, and 57.0%, respectively. We performed the same analysis for the 1416 129 alleles, and similarly created three 129 allele groups, with an average targeted segment detection efficiency of 82.6%, 70.4%, and 55.0%, respectively. We applied SnapFISH to each of the six groups and examined the identified loop candidates and loop summits. In the CAST alleles where the *Sox2* enhancer-promoter loop strength is weak due to the CAST-specific 4CBS insertion, SnapFISH can detect the loop in both “high” and “median” detection efficiency groups, but it reports 3 false positives in the “low” detection efficiency group (Supplementary Fig. S5C and Table S6). In contrast, in the 129 alleles, SnapFISH can detect the loop in all three detection efficiency groups without false positives (Supplementary Fig. S5C and Table S6). As expected, our results suggest that targeted segment detection efficiency can influence the sensitivity of detecting chromatin loops.

Additionally, we evaluated the performance of SnapFISH on an ORCA dataset^11^ generated at the same *Sox2* locus in mESCs (see details in imaging data resource). Specifically, Mateo et al. imaged the 170 Kb region around the *Sox2* gene (mm10: chr3:34,601,078-34,771,078) in mESCs, which consists of 34 5 Kb targeted segments. Across all 6007 imaged alleles, the average targeted segment detection efficiency is 56.9%. Consistent with the results in multiplexed DNA FISH data, SnapFISH identified the *Sox2* enhancer-promoter loop (Fig. 2B). Taken together, our data show that SnapFISH can accurately identify the *Sox2* enhancer-promoter loop, which had previously been identified by mESC bulk Hi-C data, from both multiplexed DNA FISH data and ORCA data.

### Applying SnapFISH to DNA seqFISH+ data in mouse excitatory neurons

We additionally re-analyzed another publicly available 25Kb resolution DNA seqFISH+ dataset from mouse cerebral cortex tissue^10^. The dataset also included RNA seqFISH data simultaneously generated from the same cells^10^, which can be used to group and annotate distinct cell clusters. We combined the three biological replicates of DNA seqFISH+ datasets to obtain a total of 2,762 cells (i.e., 5,24 alleles), with an average targeted segment detection efficiency of 40.9%. Due to the relatively low targeted segment detection efficiency, we only applied SnapFISH to the excitatory neurons^10^ consisting of 1,895 cells (i.e., 3790 alleles, with an average targeted segment detection efficiency of 43.1%), and identified 87 loops (Table S4B).

To the best of our knowledge, there is no publicly available bulk Hi-C data or single cell Hi-C data generated from excitatory neurons in mouse cerebral cortex tissue. To evaluate the functional relevance of these SnapFISH identified loops, we used recently published Paired-Tag data^35^ to obtain transcriptomic data and epigenetic data from cortical excitatory neurons L2/3 collected from mouse frontal cortex tissue (FC_L2_3 for short), including active promoter mark H3K4me3, two active enhancer marks H3K27ac and H3K4me1, and the open chromatin region mark ATAC. Figure 2C shows an illustrative example of the enhancer-promoter loop in chromosome 15, where the loop connects the promoter of gene *Myc* and the transcription end site of gene *Pvt1*. Both loop anchors overlap H3K27ac peaks and ATAC-seq peaks. We obtained similar results when applying SnapFISH to three additional mouse brain cell types (Supplementary Information Section 3 and Supplementary Fig. S6). Taken together, our results suggest that SnapFISH is able to identify putative enhancer-promoter loops from DNA seqFISH+ data in mouse brain tissue sample.

## Discussion

In this work, we report SnapFISH, the first computational pipeline to identify de novo chromatin loops from multiplexed DNA FISH data, without prior knowledge of potential loop anchor regions. We applied SnapFISH to multiplexed DNA FISH, ORCA and DNA seqFISH+ experiments in both mouse mESCs and mouse excitatory neurons, and benchmarked the performance of SnapFISH-identified chromatin loops using chromatin loops identified from bulk Hi-C data. We also showed the high reproducibility of SnapFISH between biological replicates (Supplementary Information Section 4, Supplementary Fig. S7, and Tables S7, S8), and the robustness of SnapFISH against different levels of measurement errors in the multiplexed DNA FISH experiments (Supplementary Information Section 5, Supplementary Fig. S8 and Table S9). Additionally, we provide the option of the non-parametric Wilcoxon test, as an alternative to the default two sample *T*-test (Supplementary Information Section 6 and Figs. S9, S10). SnapFISH is computationally efficient, with the computing time increasing linearly with the number of cells (Supplementary Information Section 7 and Fig. S11).

Building upon these promising results, we envision at least four directions that warrant further investigation. First of all, the sensitivity of SnapFISH can be further improved, in particular when applying to DNA seqFISH+ data in mESCs. As we showed in the analysis of multiplexed DNA FISH data in mESCs (Supplementary Fig. S5C and Table S6), a low level of targeted segment detection efficiency can reduce the sensitivity of loop detection. We expect that imputing missing 3D coordinates and missing Euclidean distance between genomic loci of interest may increase the sample size for the two sample *T*-test used in the SnapFISH algorithm (Fig. 1D), and help to enhance the statistical power of loop detection.

Second, we only considered pair-wise chromatin interactions in this work. Multiplexed DNA FISH data provide rich information on multi-way chromatin interactions, making it feasible to detect events where one enhancer interacts with multiple target genes, or one gene’s promoter interacts with multiple enhancers simultaneously. We will extend our SnapFISH framework to identify multi-way chromatin interactions, and benchmark our findings with data generated from orthogonal technologies, including immunoGAM^36^ and scSPRITE^37^.

In addition, encouraged by the success in the integrative modeling of 3D chromatin architecture datasets^38^, method developers would benefit from simulators that generate realistic multiplexed DNA FISH data, allowing flexible allocations of true loops and random collisions, precise control of the distribution of loop strength, as well as cell-to-cell variability. However, limited publicly available real data may not yet allow us to comprehensively evaluate whether data produced by a simulator is sufficiently realistic. Future studies are warranted as more data becomes available.

Last but not least, other genomic data modalities, including transcriptome and epigenome, can be imaged together with DNA in the same cell^9–12^. Integrating chromatin loops identified from SnapFISH with other genomic data modalities at single cell resolution, and characterizing their cell-to-cell variability, have the potential to reveal novel mechanisms of transcriptional regulation.

In summary, we developed SnapFISH, the first computational pipeline to identify de novo chromatin loops from multiplexed DNA FISH data. As high-resolution multiplexed DNA FISH data are increasingly available, we consider SnapFISH a valuable tool for analyzing such data, facilitating a better understanding of genome structure and genome function.

## Methods

### Definition of local neighborhood regions

For both 5 Kb bin resolution multiplexed DNA FISH data in mESCs, 5 Kb bin resolution OCRA data in mESCs, and 25 Kb bin resolution DNA seqFISH+ data in mESCs and mouse excitatory neurons, we define the local neighborhood of a given targeted segment pair as all of the identified pairs that fall within a square with 25 Kb ~ 50 Kb in 1D genomic distance from the targeted segment pair of interest. Specifically, Supplementary Fig. S1 shows the definition of local neighborhood regions, which is similar to the definition that has been used in the HiCCUPS algorithm^4^, and our recently developed SnapHiC algorithm^21^. The union of blue areas consists of the “circle” region that defines the local neighborhood.

### Two sample *T*-test

For each given targeted segment pair in each cell, we calculated the average Euclidean distance among all targeted segment pairs in its local neighborhood regions, as used such average Euclidean distance as the control. Due to the missing data in multiplexed DNA FISH data, not all targeted segments are observed. Therefore, we applied two sample *T*-test, instead of paired *T*-test, to evaluate the statistical significance of the difference in Euclidean distance between a given targeted segment pair and its local neighborhood. In addition, SnapFISH provided the non-parametric Wilcoxon test, as an alternative to the default two sample *T*-test.

### Identification of loop candidates

We define a pair of segments as a loop candidate if and only its average Euclidean distance is smaller than that of the average Euclidean distance for segment pairs in the local neighborhood (T < 0) and FDR < 10% (Fig. 1E).

### Identification of loop summits

We group nearby loop candidates within a pre-specified gap into clusters, where the gap is twice the size of the bin resolution. In other words, we define the gap to be 10 Kb for 5 Kb resolution multiplexed DNA FISH data and 5 Kb resolution OCRA data, and 50 Kb for 25 Kb resolution DNA seqFISH+ data. Among each cluster, we select the pair of targeted segments with the minimal FDR as the summit. The final loop list consists of cluster summits with population-level contact frequency >=1/3, and singletons with population-level contact frequency >=1/2. Detailed justification of threshold values can be found in Supplementary Information Section 1.

### Overlap of SnapFISH-identified loop with *Sox2* enhancer-promoter loop

We defined chr3:34,645,000–34,655,000 and chr3:34,755,000–34,765,000 as the two 10 Kb bins containing *Sox2* promoter and super-enhancer, respectively. We further defined a SnapFISH-identified 5 Kb bin resolution loop as “overlapped” with the *Sox2* enhancer-promoter, if and only if the SnapFISH loop anchors are within 10 Kb of *Sox2* promoter-super-enhancer loop anchors.

### Statistics and reproducibility

In this study, we re-analyzed the publicly available datasets (see details in the “Data Availability” section below). No statistical method was used to predetermine sample size. No data were excluded from the analyses. The experiments were not randomized. The investigators were not blinded to allocating during experiments and outcome assessment. In addition, we evaluated the reproducibility of SnapFISH among biological replicates (see details in Supplementary Information Section 4).

### Reporting summary

Further information on research design is available in the Nature Portfolio Reporting Summary linked to this article.

### Supplementary information

Supplementary Information

Peer Review File

Description of Additional Supplementary Files

Supplementary Dataset 1

Supplementary Dataset 2

Reporting Summary

### Source data

Source Data

## Data Availability

All relevant data supporting the key findings of this study are available within the article and its Supplementary Information files. Imaging data resource. 25 Kb bin resolution DNA seqFISH+ data from mESC. We downloaded DNA seqFISH+ data from the website https://zenodo.org/record/3735329, and used two files (DNAseqFISH+25kbloci-E14-replicate1.csv and DNAseqFISH+25kbloci-E14-replicate2.csv). Such data are originally used in the Takei et al. study^9^. 5 Kb bin resolution multiplexed DNA FISH data from mESC at the *Sox2* gene locus. We downloaded multiplexed DNA FISH data from the 4D Nucleome data portal (https://data.4dnucleome.org/experiment-set-replicates/4DNESC5PKTQ9/), which were originally used in the Huang et al. study^18^. 5 Kb bin resolution ORCA data from mESC at the *Sox2* locus. ORCA data are shared by Dr. Boettiger, which were originally used in the Mateo et al. study^11^. We shared ORCA data as the file “Supplementary Dataset 1.csv” in this paper. 25 Kb bin resolution DNA seqFISH+ data from mouse cerebral cortex tissue. We downloaded DNA seqFISH+ data from the website https://zenodo.org/record/4708112, and used the file (TableS8_brain_DNAseqFISH_25kb_voxel_coordinates_2762cells.csv). Such data are originally used in the Takei et al. study^10^. 2. mESC ChIP-seq data resource mESC H3K4me3 ChIP-seq data is from our previous study^32^. mESC H3K27ac ChIP-seq data is downloaded from the ENCODE website: https://www.encodeproject.org/experiments/ENCSR000CGQ/. mESC CTCF ChIP-seq data is downloaded from the ENCODE website: https://www.encodeproject.org/experiments/ENCSR000CCB/. Reference genomes We used mm10 for imaging data generated from mESCs and mouse excitatory neurons. Source data are provided with this paper.

## Code availability

SnapFISH is freely available at https://github.com/HuMingLab/SnapFISH. SnapFISH source code^39^ is available at https://zenodo.org/record/8083569.
