# Supplementary material for: SnapFISH: a computational pipeline to identify chromatin loops from multiplexed DNA FISH data
Source: Nat Commun. 2023 Aug 12;14:4873. doi: 10.1038/s41467-023-40658-3 (PMC10423204; doi:10.1038/s41467-023-40658-3)

Title page and index for the Supplementary Information

Index of Supplementary information: page 1

Supplementary text Section 1: page 2

Supplementary text Section 2: page 2

Supplementary text Section 3: page 3

Supplementary text Section 4: page 3

Supplementary text Section 5: page 4

Supplementary text Section 6: page 5

Supplementary text Section 7: page 5

Supplementary references: page 6

Figure S1: page 7

Figure S2: page 8

Figure S3: page 14

Figure S4: page 19

Figure S5: page 21

Figure S6: page 25

Figure S7: page 26

Figure S8: page 29

Figure S9: page 32

Figure S10: page 33

Figure S11: page 34

Supplementary Information

Section 1: Necessity of developing tailored loop caller for multiplexed DNA FISH data.

Many computational pipelines have been developed for identifying chromatin loops from Hi-C datasets, including HiCCUPS[1], FitHiC[2]/FitHiC2[3], SIP[4], Peakachu[5], etc (see detailed benchmark analysis in the reference[6]). However, none of these methods can be directly applied to multiplexed DNA FISH data due to two major reasons. First, all existing Hi-C loop callers are based on statistical modeling of count data. The underlying Poisson or negative binomial model does not fit the continuous data measuring Euclidean distance in multiplexed DNA FISH data. Although researchers could convert multiplexed DNA FISH data into count-based data similar to Hi-C, by first pre-specifying a Euclidean distance threshold (as described in a recent preprint[7]), and counting the number of cells with Euclidean distance smaller than the pre-specified threshold. However, due to missing loci in multiplexed DNA FISH data, the number of available cells at each loci pair is different. Therefore, one can only use the frequency, instead of the count, for downstream loop calling analysis, making the count-based Poisson or negative binomial model inappropriate.

To fill in such gap, we developed SnapFISH, a loop caller tailored for multiplexed DNA FISH data. Different from all existing Hi-C-based loop callers, SnapFISH uses two sample T-test to model the continuous Euclidean distance, providing rigorous statistical justification of the loop calling pipeline. SnapFISH also provides the non-parametric Wilcoxon rank test as an alternative, when the assumption of T-test is violated (see details in **Supplementary Information Section 6**). In addition, SnapFISH applies the contact frequency filtering based on the average Euclidean distance between two loci with 25Kb 1D genomic distance (see details in **Supplementary Information Section 2**), achieving high precision and satisfactory recall (**Table S3**). Taken together, the methodological innovations in SnapFISH make it a tailored loop caller for multiplexed DNA FISH data, compared to the existing Hi-C-based loop callers.

Section 2: Optimizing the SnapFISH algorithm using DNA seqFISH+ data in mESCs.

We evaluated different combinations of SnapFISH parameters, in order to achieve optimal F1-score, benchmarking against the reference loop set consisting of 35 HiCCUPS loops identified from deeply sequenced bulk Hi-C data. First of all, we performed both two sample T-test and the non-parametric Wilcoxon test. For both two tests, we converted P-values into false discovery rate (FDR), and evaluated 3 FDR threshold values (FDR<10%, FDR<5% and FDR<1%) to define loop candidates. We then grouped nearby loop candidates into clusters, and identified cluster summits and singletons. We evaluated two approaches to select summits: using the loci pair with the smallest FDR, or using the loci pair nearest to the geometric center of the cluster. In the end, the final loop list consists of cluster summits with population-level contact frequency >= Cutoff.summit and singletons with population-level contact frequency >= Cutoff.singleton. We applied a grid search to evaluate all combinations of Cutoff.summit and Cutoff.singleton, with the step size of 1/6 and under the constraint that Cutoff.summit <= Cutoff.singleton. We only calculated F1-score for the combination of SnapFISH parameters resulting in >=1 loop summit. Taken together, we evaluated 144 combinations of SnapFISH parameters, and selected the one corresponding to the highest F1-score (**Table S3**). With the optimized SnapFISH parameters, we used two sample T-test FDR<10% to select loop candidates, and the final loop list contained cluster summits (with the minimal FDR) with population-level contact frequency >= 1/3, and singletons with population-level contact frequency >= 1/2. SnapFISH identified 16 loops, where 14 loops overlapped HiCCUPS loops. The precision, recall and F1-score were 87.5%, 40.0% and 0.549, respectively.

In addition, we evaluated the performance of SnapFISH with different sizes of the local neighborhood control regions. As the default setting, SnapFISH defines the local neighborhood control regions as the loci pairs within 1D genomic distance 25Kb~50Kb to the loci pair of interest, for both 5Kb and 25Kb bin resolution multiplexed DNA FISH data (**Figure S1**), and such a distance threshold is motivated by the HiCCUPS[1] algorithm and our SnapHiC[8] algorithm. In this analysis, we consider 3 additional threshold values for 1D genomic distance: 0~25Kb, 50Kb~75Kb and 75Kb~100Kb. When using loci pairs with 1D genomic distance 0~25Kb as the control, SnapFISH did not find any loop candidate, since the minimal FDR based on two sample T-test is 0.1237, underscoring the necessity of removing immediately adjacent loci pairs in the local neighborhood control region. When using loci pairs with 50Kb~75Kb as the control, SnapFISH identified 18

loops with 10 true positives. The precision, recall and F1-score are 55.6%, 28.6% and 0.377, respectively. When using loci pairs with 75Kb~100Kb as the control, SnapFISH identified 11 loops with 6 true positives. The precision, recall and F1-score are 54.6%, 17.1% and 0.261, respectively. Taken together, our data suggest that the default setting with 1D genomic distance 25Kb ~ 50Kb achieves the best performance with the highest F1-score.

The current version of SnapFISH only allows 5Kb bin and 25Kb bin resolutions, and our results demonstrate that SnapFISH works well for all the datasets used in this study. We recommend using the default setting for bin resolution between 5Kb and 25Kb. For finer resolution data (i.e., 2Kb), future work is warranted to optimize SnapFISH parameters, including both the size of local neighborhood control regions, statistical significance levels and contact frequency thresholds. We will pursue this research direction when more genome-wide finer resolution imaging data become publicly available.

Section 3: Applying SnapFISH to DNA seqFISH+ data in three additional mouse brain cell types.

Takei et al study[9] contains DNA seqFISH+ data of 2,762 cells from nine major cell type clusters. We have shown that SnapFISH can identify enhancer-promoter loops in mouse excitatory neurons (**Figure 2C**), which is the largest cell type cluster consisting of 1,895 cells (3,790 alleles). We then evaluated the performance of SnapFISH on other cell type clusters with a smaller number of cells. Since our previous analysis has shown that SnapFISH can only identify the *Sox2* enhancer-promoter loop with more than 200 alleles (**Figure S5** and **Table S5**), we applied SnapFISH to DNA seqFISH+ data generated from three additional major cell type clusters with more than 200 alleles, including inhibitory neurons expressing parvalbumin (Pvalb for short, 155 cells, 310 alleles), astrocytes (Astro for short, 152 cells, 304 alleles) and endothelial cells (Endo for short, 240 cells, 480 alleles). All the remaining five cell type clusters consist of less than 200 alleles.

Notably, the targeted segment detection efficiency is 39.5%, 38.6% and 19.6% for Pvalb, Astro and Endo, respectively, which are comparable (Pvalb and Astro) or much lower (Endo) than the 43.1% targeted segment detection efficiency in excitatory neurons. The low targeted segment detection efficiency and the small number of alleles in these three cell types may result in the limited sensitivity of loop calling. As expected, SnapFISH identified 5, 5 and 3 loops in Pvalb, Astro and Endo, respectively (**Table S4C**), which are much less than the 87 loops identified from excitatory neurons. We also used Paired-Tag data[10] to annotate these SnapFISH-identified loops. **Figure S6** showed an illustrative example of an enhancer-promoter loop in chromosome 3 in Astro, where the loop connects the promoter of gene *Adra1a* to an ATAC peak ~200Kb upstream. This example showcased that SnapFISH can identify enhancer-promoter loops in Astro with much smaller cell number than excitatory neurons.

Section 4. Evaluation the reproducibility of SnapFISH among biological replicates.

We first evaluated the reproducibility of SnapFISH using 25Kb bin resolution DNA seqFISH+ data generated from mESCs[11], which consists of two biological replicates. Replicate #1 contains 201 cells (i.e., 402 alleles) with an average targeted segment detection efficiency of 58.6%, while Replicate #2 contains 245 cells (i.e., 490 alleles) with an average targeted segment detection efficiency of 70.6%. Applying SnapFISH to these two replicates separately identified 6 loops and 14 loops in Replicate #1 and Replicate #2, respectively (**Table S7**). The more loops detected from Replicate #2 might be due to the fact that Replicate #2 contains more cells with a higher targeted segment detection efficiency. Reassuringly, all 6 loops identified from Replicate #1 overlap with loops identified from Replicate #2 (**Table S7**). As one illustrative example in chromosome 16, **Figure S7A** shows that the loop identified exclusively by SnapFISH in Replicate #1 is reproducible in Replicate #2. Notably, SnapFISH-identified loops in both Replicate #1 and Replicate #2 overlap HiCCUPS-identified loops from mESC bulk Hi-C data (**Figure S7A**).

We further evaluated the reproducibility of SnapFISH using 25Kb bin resolution DNA seqFISH+ data generated from mouse excitatory neurons[9], which consists of three biological replicates. Replicate #1 contains 1,077 alleles with an average targeted segment detection efficiency of 41.7%, Replicate #2 contains 1,072 alleles with an average targeted segment detection efficiency of 42.8%, while Replicate #3 contains 1,567 alleles with an average targeted segment detection efficiency of 46.2%. Applying SnapFISH to these three replicates

separately identified 30 loops, 59 loops and 71 loops in Replicate #1, Replicate #2 and Replicate #3, respectively (**Table S8**). For each replicate, we defined reproducibility as the number of loops overlapped (up to 50Kb gap) in at least one of two other replicates. Based on such definition, 23 out of 30 loops in Replicate #1 (76.7%), 34 out of 59 loops in Replicate #2 (57.6%) and 39 out of 71 loops in Replicate #3 (54.9%) are reproducible (**Table S8**). As one illustrative example in chromosome 16, **Figure S7B** shows that SnapFISH-identified loops are reproducible among 3 replicates, while SnapFISH identified more loops from Replicate #3 with more alleles and higher detection efficiency than in Replicate #1 and Replicate #2.

Taken together, our data showed that SnapFISH can identify reproducible loops among different biological replicates in both mESCs and mouse excitatory neurons. The loops specific to only one replicate may be due to the high cell-to-cell variability of chromatin loops among the cell population.

Section 5. Evaluation the robustness of SnapFISH against different levels of measurement errors in multiplexed DNA FISH experiments.

SnapFISH takes the (X, Y, Z) coordinates measured in multiplexed DNA FISH experiments as the input, and generates a list of chromatin loops. However, those (X, Y, Z) coordinates do not reflect precise 3D positions, but rather positions estimated from Gaussian approximation or other fitting approaches. Here we performed benchmark analysis to evaluate the robustness of the SnapFISH loop calling again different levels of measurement errors in multiplexed DNA FISH experiments.

First of all, we collected 5Kb resolution ORCA data at the mESC *Sox2* locus with measurement error, which contains the 95% confidence interval (CI) for each (X, Y, Z) coordinates. We calculated the average length of 95% CI for each allele, and divided all 6,007 alleles into three groups: low uncertainty (2,002 alleles, length of 95% CI <17.21nm, detection efficiency 64.8%), median uncertainty (2,002 alleles, 17.21nm <= length of 95% CI <20.56nm, detection efficiency 60.9%) and high uncertainty (2,003 alleles, length of 95% CI >=20.56nm, detection efficiency 45.0%). For all 3 groups of measurement errors, SnapFISH can accurately identify the *Sox2* enhancer-promoter loop (**Figure S8A** and **Table S9A**). Notably, in the low and median uncertainty groups, SnapFISH identified one additional CTCF-CTCF loop, which overlaps chromatin interactions identified from mESC CTCF PLAC-seq data[12].

To the best of our knowledge, none of the two publicly available DNA seqFISH+ datasets[9,11] nor chromatin tracing data[13] contains uncertainty measurement. Therefore, we performed a simulation-based robustness analysis as follows.

We first added simulated measurement errors into the chromatin tracing data at the mESC *Sox2* locus. Previous study[14] has shown that measurement errors in chromatin tracing data are ~40nm for the X and Y coordinates, and ~50nm for the Z coordinate. Therefore, we simulated measurement errors from Gaussian distribution with mean 0 and three different standard deviations (sd): low uncertainty (sd=25nm), median uncertainty (sd=50nm) and high uncertainty (sd=100nm). We then added the simulated measurement error to the (X, Y, Z) coordinates of 1,416 129 alleles. For all 3 types of measurement errors, SnapFISH can accurately identify the *Sox2* enhancer-promoter loop (**Figure S8B** and **Table S9B**).

Moreover, we simulated measurement errors into the 25Kb resolution DNA seqFISH+ data from mESCs. Similar to the abovementioned analysis for chromatin tracing data, we added three types of measurement errors, and benchmarked the performance of SnapFISH on the perturbed data (**Table S9C**). Specifically, when adding low level of measurement errors (i.e., sd=25nm), SnapFISH identified 18 loops, among which 14 are true positive. The precision, recall and F1-score are 77.8%, 40.0% and 0.528, respectively. When adding median level of measurement errors (i.e., sd=50nm), SnapFISH identified 19 loops, among which again 14 loops are true positive. The precision, recall and F1-score are 73.7%, 40.0% and 0.519, respectively. When adding high level of measurement errors (i.e., sd=100nm), SnapFISH identified 10 loops, and 8 loops are true positive. The precision, recall and F1-score are 80.0%, 22.9% and 0.356, respectively.

Taken together, our results show that SnapFISH can robustly identify the *Sox2* enhancer-promoter loop at different levels of measurement errors for both ORCA data and chromatin tracing data. For the genome-wide benchmark analysis using mESC DNA seqFISH+ data, adding low to median level of measurement error leads to a slight increase of false positive (i.e., a decrease in precision). However, adding high level of measurement error leads to a decrease in precision and large reduction in sensitivity. Collectively, our results demonstrate that SnapFISH is robust to different levels of measurement errors in multiplexed DNA FISH data.

Section 6. Using non-parametric Wilcoxon test as an alternative to the default two-sample T-test.

The distribution of Euclidean distance between a pair of loci across cells may deviate substantially from the normal distribution (e.g., heavily skewed, or having multiple modes). We therefore provide the non-parametric Wilcoxon test as an alternative to the default two-sample T-test. In our previous analysis (**Table S3**), we have shown that the Wilcoxon test and T-test provide similar genome-wide loop calling results in mESC DNA seqFISH+ data. As one illustrative example, we evaluated the *Sox2* enhancer-promoter loop using the mESC chromatin tracing data.

Specifically, we first plotted the distribution of Euclidean distance between the *Sox2* promoter and its super-enhancer, and the average Euclidean distance for loci pairs in the local neighborhood control regions, in both CAST allele and 129 allele (**Figure S9A** and **Figure S9B**). The distance distribution is approximately normal, supporting the use of T-test. In the CAST allele (**Figure S9A**), the p-value from two-sided two sample T-test and the two-sided two sample Wilcoxon rank test are 5.74e-20 and 2.86e-40, respectively. In the 129 allele (**Figure S9B**), the p-value from two-sided two sample T-test and the two-sided two sample Wilcoxon rank test are 2.51e-13 and 1.52e-38, respectively. To evaluate why the Wilcoxon rank test generates more significant p-values than T-test, we used the alleles without missing data at the *Sox2* enhancer-promoter loop (N=469 for the CAST allele, N=498 for the 129 allele), and plotted the Euclidean distance of the *Sox2* enhancer-promoter loop against the average Euclidean distance for loci pairs in the local neighborhood control regions (**Figure S10A** and **Figure S10B**). In both CAST and 129 alleles, the majority of alleles showed slightly smaller Euclidean distance at the *Sox2* enhancer-promoter loop compared to that in its local neighborhood control regions. The majority imbalanced towards one direction but with relatively small differences together explain the more significant p-values from the Wilcoxon rank test than from T-test.

Section 7. Time and memory cost of SnapFISH.

We evaluated the time and memory cost of SnapFISH, using 5Kb bin resolution mESC multiplexed DNA FISH data with different numbers of the CAST alleles and the 129 alleles (i.e., # of alleles = 100, 200, 300, 400 and 500). **Figure S11** showed that SnapFISH is computationally efficient, which only takes ~10 seconds to analyze 500 alleles, and the running time increases approximately linearly with the number of alleles. In addition, SnapFISH requires ~120Mb memory for different numbers of CAST alleles and 129 alleles. All computation tasks are performed in the single compute node with a 2.40 GHz Intel processor and 256 Gb RAM.

References

1 Rao, Suhas S. P. *et al.* A 3D Map of the Human Genome at Kilobase Resolution Reveals Principles of Chromatin Looping. *Cell* **159**, 1665-1680 (2014).

2 Ay, F., Bailey, T. L. & Noble, W. S. Statistical confidence estimation for Hi-C data reveals regulatory chromatin contacts. *Genome Res* **24**, 999-1011 (2014). https://doi.org:10.1101/gr.160374.113

3 Kaul, A., Bhattacharyya, S. & Ay, F. Identifying statistically significant chromatin contacts from Hi-C data with FitHiC2. *Nat Protoc* **15**, 991-1012 (2020). https://doi.org:10.1038/s41596-019-0273-0

4 Rowley, M. J. *et al.* Analysis of Hi-C data using SIP effectively identifies loops in organisms from C. elegans to mammals. *Genome Res* **30**, 447-458 (2020). https://doi.org:10.1101/gr.257832.119

5 Salameh, T. J. *et al.* A supervised learning framework for chromatin loop detection in genome-wide contact maps. *Nat Commun* **11**, 3428 (2020). https://doi.org:10.1038/s41467-020-17239-9

6 Forcato, M. *et al.* Comparison of computational methods for Hi-C data analysis. *Nat Methods* **14**, 679-685 (2017). https://doi.org:10.1038/nmeth.4325

7 Hafner, A., Park, M., Berger, S. E., Nora, E. P. & Boettiger, A. N. Loop stacking organizes genome folding from TADs to chromosomes. *bioRxiv*, 2022.2007.2013.499982 (2022). https://doi.org:10.1101/2022.07.13.499982

8 Yu, M. *et al.* SnapHiC: a computational pipeline to identify chromatin loops from single-cell Hi-C data. *Nat Methods* **18**, 1056-1059 (2021). https://doi.org:10.1038/s41592-021-01231-2

9 Takei, Y. *et al.* Single-cell nuclear architecture across cell types in the mouse brain. *Science* **374**, 586-594 (2021). https://doi.org:10.1126/science.abj1966

10 Zhu, C. *et al.* Joint profiling of histone modifications and transcriptome in single cells from mouse brain. *Nat Methods* **18**, 283-292 (2021). https://doi.org:10.1038/s41592-021-01060-3

11 Takei, Y. *et al.* Integrated spatial genomics reveals global architecture of single nuclei. *Nature* **590**, 344-350 (2021). https://doi.org:10.1038/s41586-020-03126-2

12 Juric, I. *et al.* MAPS: Model-based analysis of long-range chromatin interactions from PLAC-seq and HiChIP experiments. *PLoS Comput Biol* **15**, e1006982 (2019). https://doi.org:10.1371/journal.pcbi.1006982

13 Huang, H. *et al.* CTCF mediates dosage- and sequence-context-dependent transcriptional insulation by forming local chromatin domains. *Nat Genet* **53**, 1064-1074 (2021). https://doi.org:10.1038/s41588-021-00863-6

14 Bintu, B. *et al.* Super-resolution chromatin tracing reveals domains and cooperative interactions in single cells. *Science* **362** (2018). https://doi.org:10.1126/science.aau1783

Figure S1. Cartoon illustration of local neighborhood control region. **A.** 5Kb bin resolution for multiplexed DNA FISH data and ORCA data. **B.** 25Kb bin resolution for DNA seqFISH+ data. In both, the red area represents the pair of targeted segments of interest. The blue area represents the local neighborhood control region defined as a circle with 25Kb~50Kb 1D genomic distance (see details in **Methods**). In both, the grey areas are regions within 25Kb 1D genomic distance of the pair of targeted segments of interest, which are excluded from the definition of local neighborhood control region. Source data are provided.

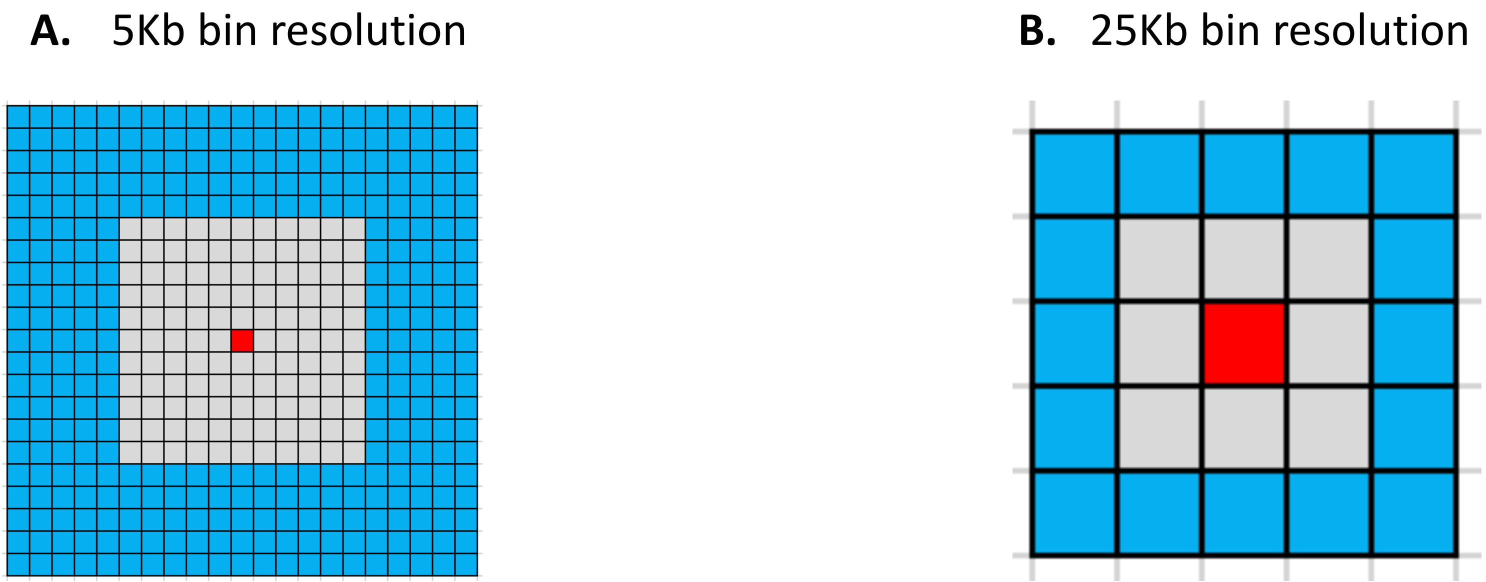

Figure S2. Heatmap of average Euclidean distance (unit: nm) and population-level contact frequency from mESC DNA seqFISH+ data, and heatmap of Hi-C contact frequency (unit: log2 KR-normed Hi-C contact frequency) from mESC bulk Hi-C data. In each sub-figure, the left and middle panel is the heatmap of average Euclidean distance and contact frequency from mESC DNA seqFISH+ data, respectively. The right panel is the heatmap of Hi-C contact frequency from mESC bulk Hi-C data. We showed 8 chromosomes that contain 10Kb bin resolution and 25Kb bin resolution HiCCUPS-identified loops: **A.** chromosome 2 (1 SnapFISH loop, 4 HiCCUPS loops). **B.** chromosome 3 (6 SnapFISH loops, 4 HiCCUPS loops). **C.** chromosome 6 (4 SnapFISH loops, 7 HiCCUPS loops). **D.** chromosome 9 (no SnapFISH loop, 4 HiCCUPS loops). **E.** chromosome 10 (no SnapFISH loop, 4 HiCCUPS loops). **F.** chromosome 11 (no SnapFISH loop, 1 HiCCUPS loop). **G.** chromosome 15 (1 SnapFISH loop, 7 HiCCUPS loops). **H.** chromosome 16 (4 SnapFISH loops, 4 HiCCUPS loops). Note that imaging data in chromosome 2 (**A.**), chromosome 3 (**B.**) and chromosome 6 (**C.**) contain missing data, which are shown in grey vertical and horizontal lines. In each heatmap, the dashed black circles (with the grey pixel in the middle) in the lower left triangle represent SnapFISH-identified loops (left and middle), or HiCCUPS-identified loops (right). **I.** Population-level contact frequency for 14 true positives (HiCCUPS loops identified by SnapFISH) and 21 false negatives (HiCCUPS loops missed by SnapFISH). In each box, the upper edge, horizontal center line and lower edge represent the 75th percentile, median and 25th percentile, respectively. The upper whiskers represent the 75th percentile + 1.5× the interquartile range (IQR). The lower whiskers represent the 25th percentile - 1.5× the interquartile range (IQR). Source data are provided.

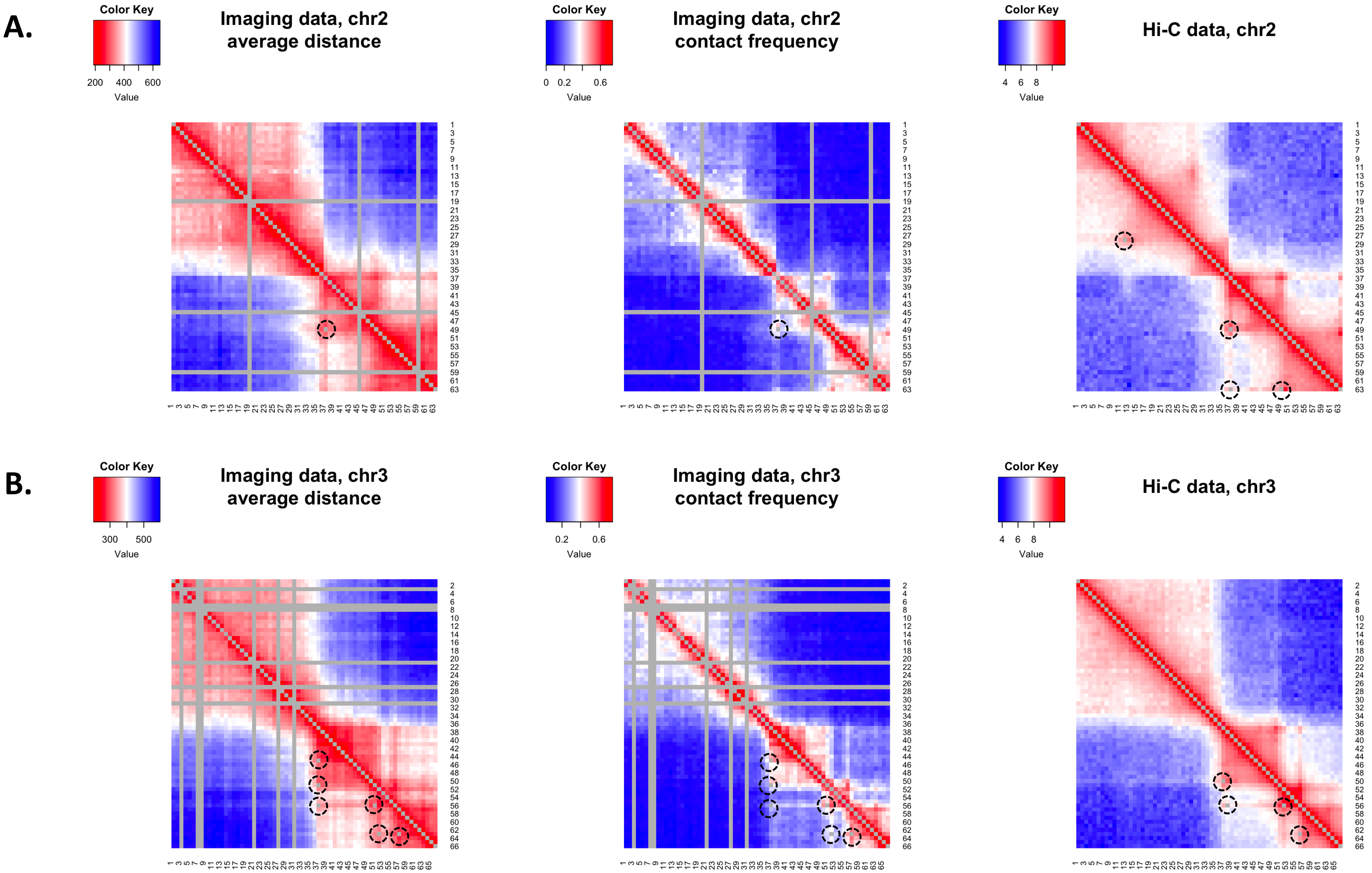
A.
Color Key
200 400 600
Value
Imaging data, chr2
average distance
Color Key
0 0.2 0.6
Value
Imaging data, chr2
contact frequency
Color Key
4 6 8
Value
Hi-C data, chr2
B.
Color Key
300 500
Value
Imaging data, chr3
average distance
Color Key
0.2 0.6
Value
Imaging data, chr3
contact frequency
Color Key
4 6 8
Value
Hi-C data, chr3

C.

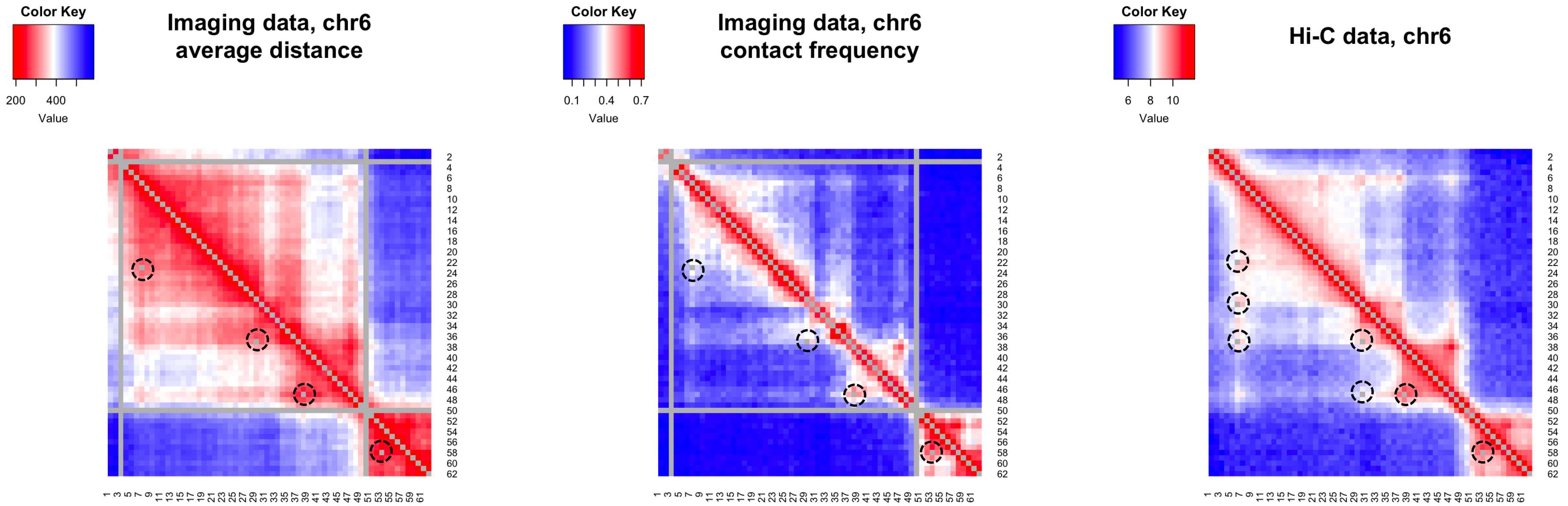
Color Key
200 400
Value
Imaging data, chr6
average distance
Color Key
0.1 0.4 0.7
Value
Imaging data, chr6
contact frequency
Color Key
6 8 10
Value
Hi-C data, chr6

D.

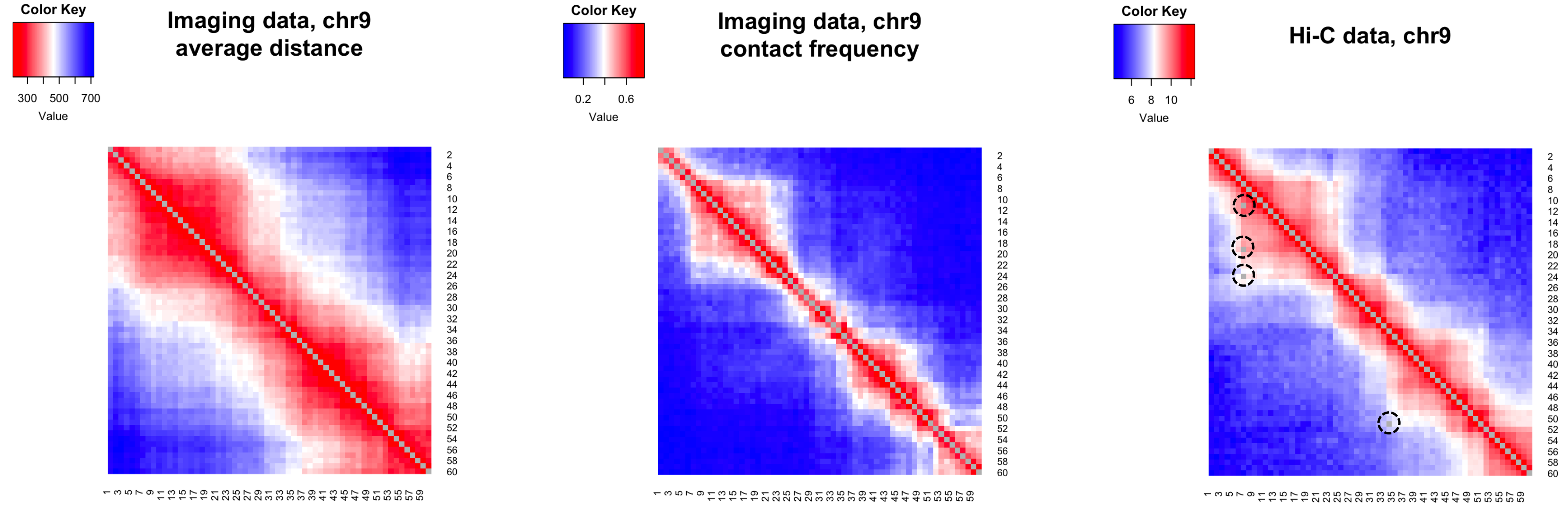
Color Key
300 500 700
Value
Imaging data, chr9
average distance
Color Key
0.2 0.6
Value
Imaging data, chr9
contact frequency
Color Key
6 8 10
Value
Hi-C data, chr9

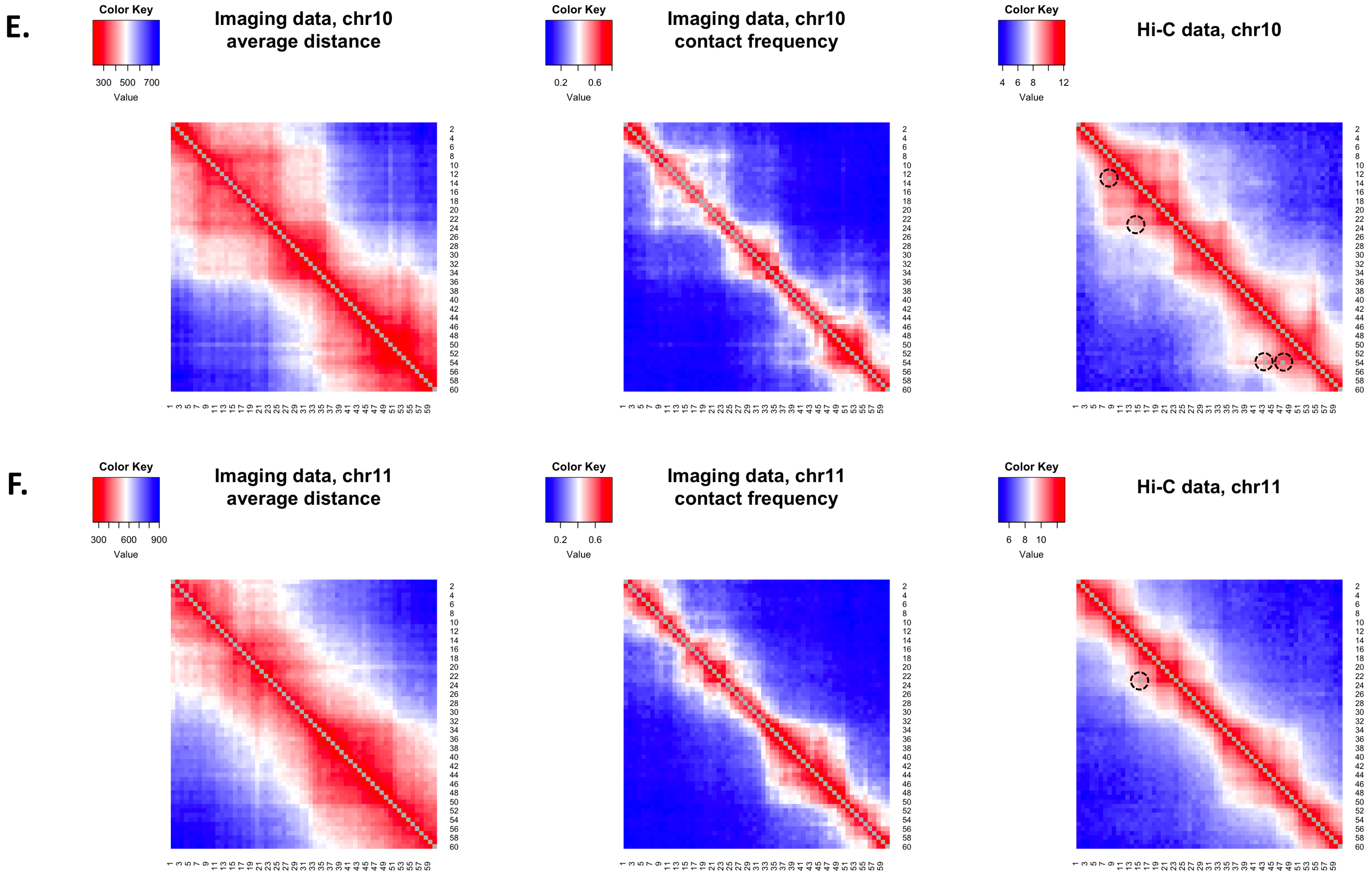
E.
Color Key
300 500 700
Value
Imaging data, chr10
average distance
Color Key
0.2 0.6
Value
Imaging data, chr10
contact frequency
Color Key
4 6 8 12
Value
Hi-C data, chr10
F.
Color Key
300 600 900
Value
Imaging data, chr11
average distance
Color Key
0.2 0.6
Value
Imaging data, chr11
contact frequency
Color Key
6 8 10
Value
Hi-C data, chr11

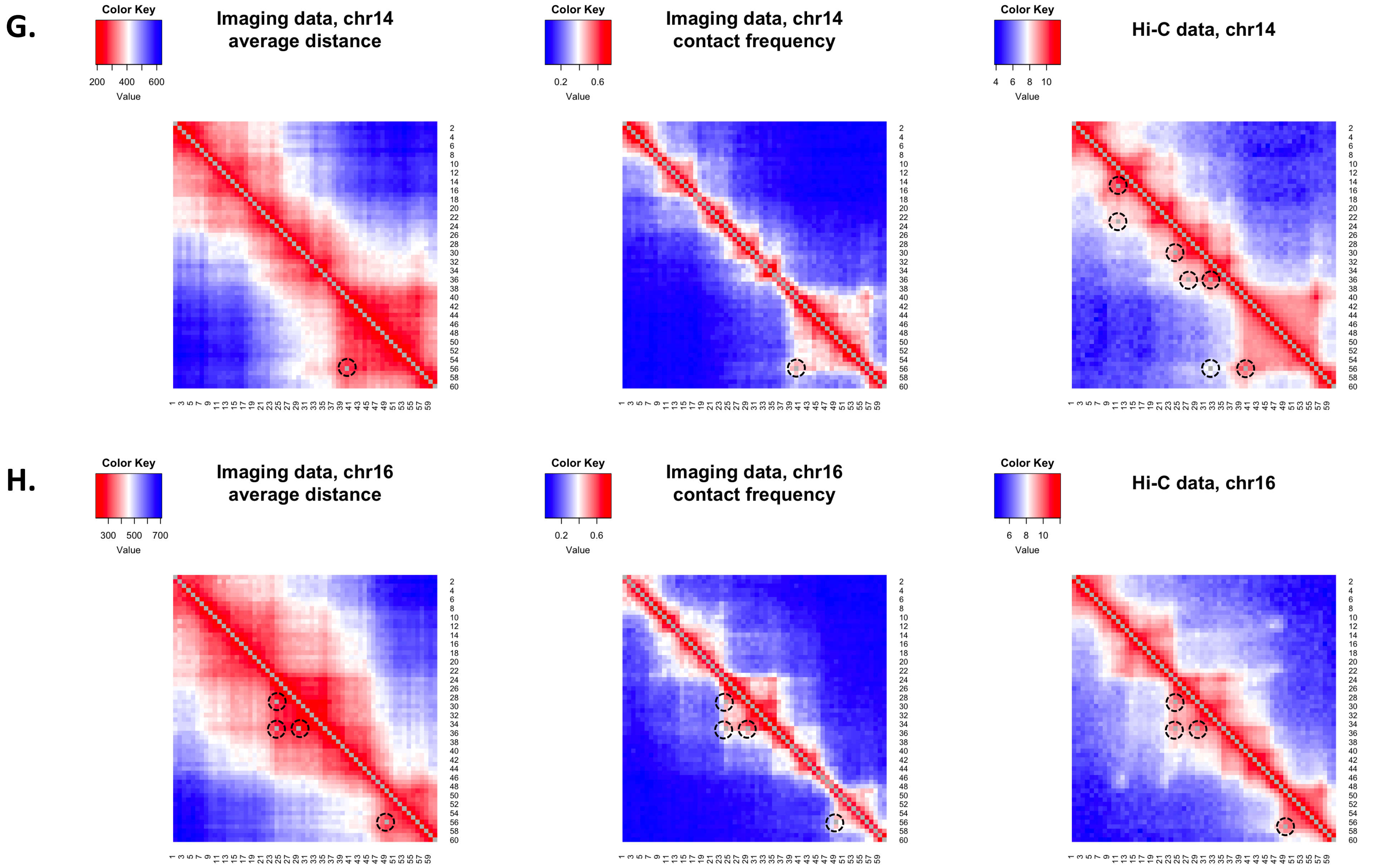
G.
Color Key
200 400 600
Value
Imaging data, chr14
average distance
Color Key
0.2 0.6
Value
Imaging data, chr14
contact frequency
Color Key
4 6 8 10
Value
Hi-C data, chr14
H.
Color Key
300 500 700
Value
Imaging data, chr16
average distance
Color Key
0.2 0.6
Value
Imaging data, chr16
contact frequency
Color Key
6 8 10
Value
Hi-C data, chr16

I.

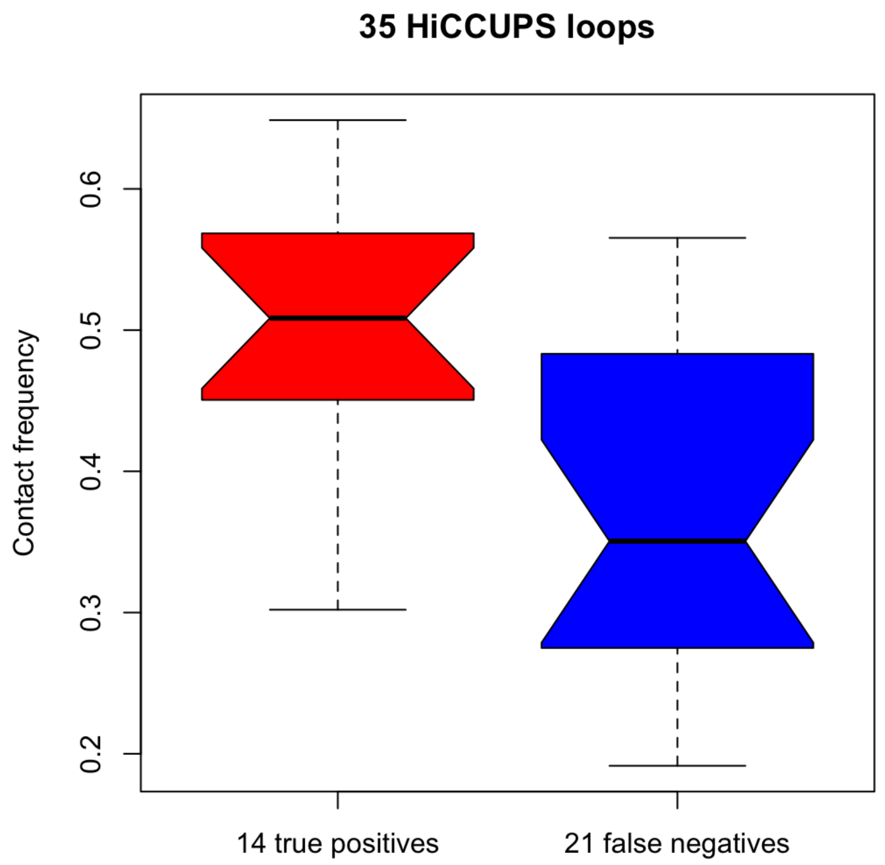
35 HiCCUPS loops
Contact frequency
0.2
0.3
0.4
0.5
0.6
14 true positives
21 false negatives

Figure S3. SnapFISH identified loops with high accuracy using DNA seqFISH data from mESC. Only chromosomes with SnapFISH-identified loops are shown. In each panel, the top three tracks are mESC CTCF, mESC H3K4me3 and mESC H3K27ac ChIP-seq data. The middle three tracks represent the HiCCUPS-identified loop at 25Kb bin resolution, the HiCCUPS-identified loop at 10Kb bin resolution, and the SnapFISH-identified loop at 25Kb bin resolution, respectively. The bottom track is the Refseq gene annotation. **A.** 6 SnapFISH-identified loops in chromosome 3. **B.** 4 SnapFISH-identified loops in chromosome 6. **C.** 1 SnapFISH-identified loop in chromosome 14. **D.** 4 SnapFISH-identified loop in chromosome 16. Source data are provided.

A. Six SnapFISH-identified loops in chromosome 3. Two false positives are CTCF-CTCF loop (left) and enhancer/promoter-promoter loop (right).

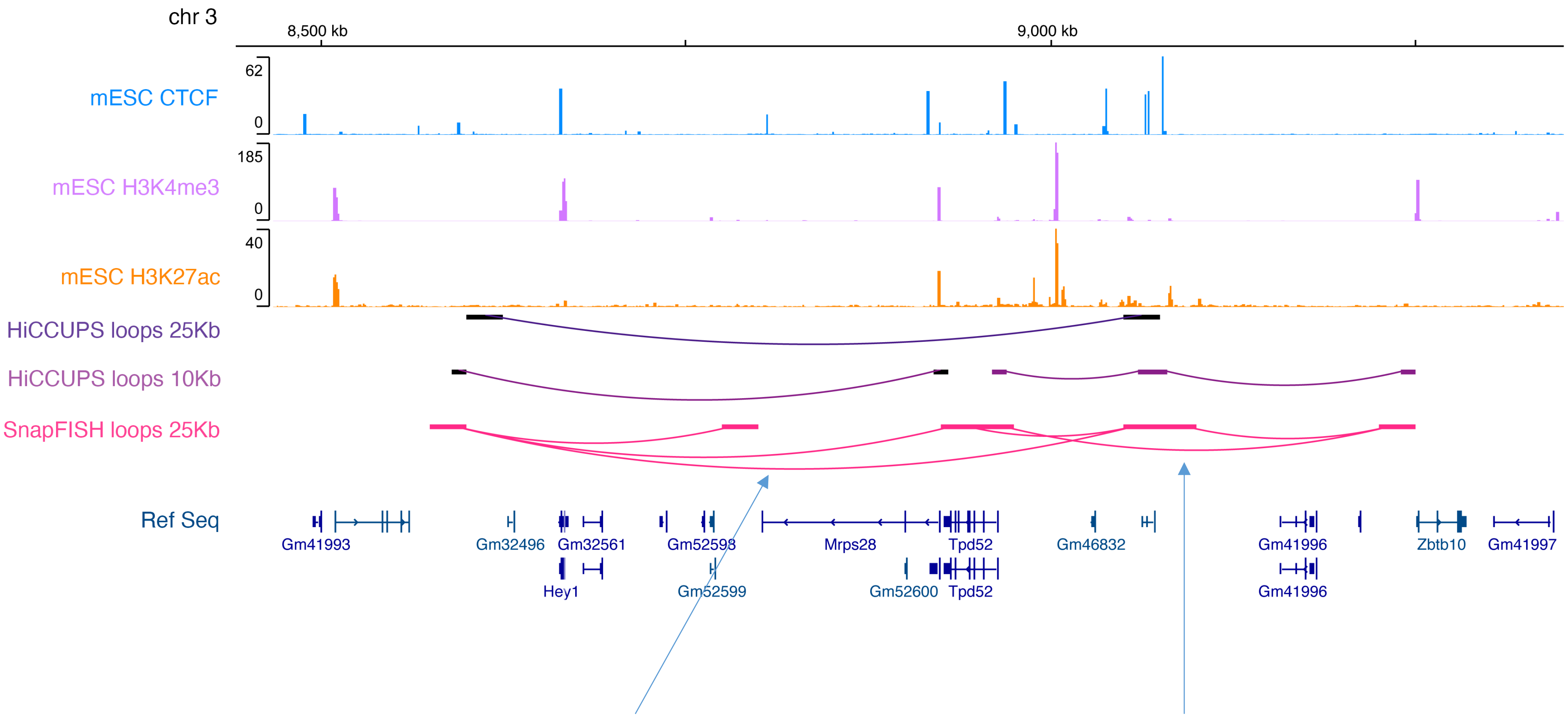

False positive #1:
CTCF-CTCF loop

False positive #2:
Enhancer/promoter-promoter loop

B. Four SnapFISH-identified loops in chromosome 6.

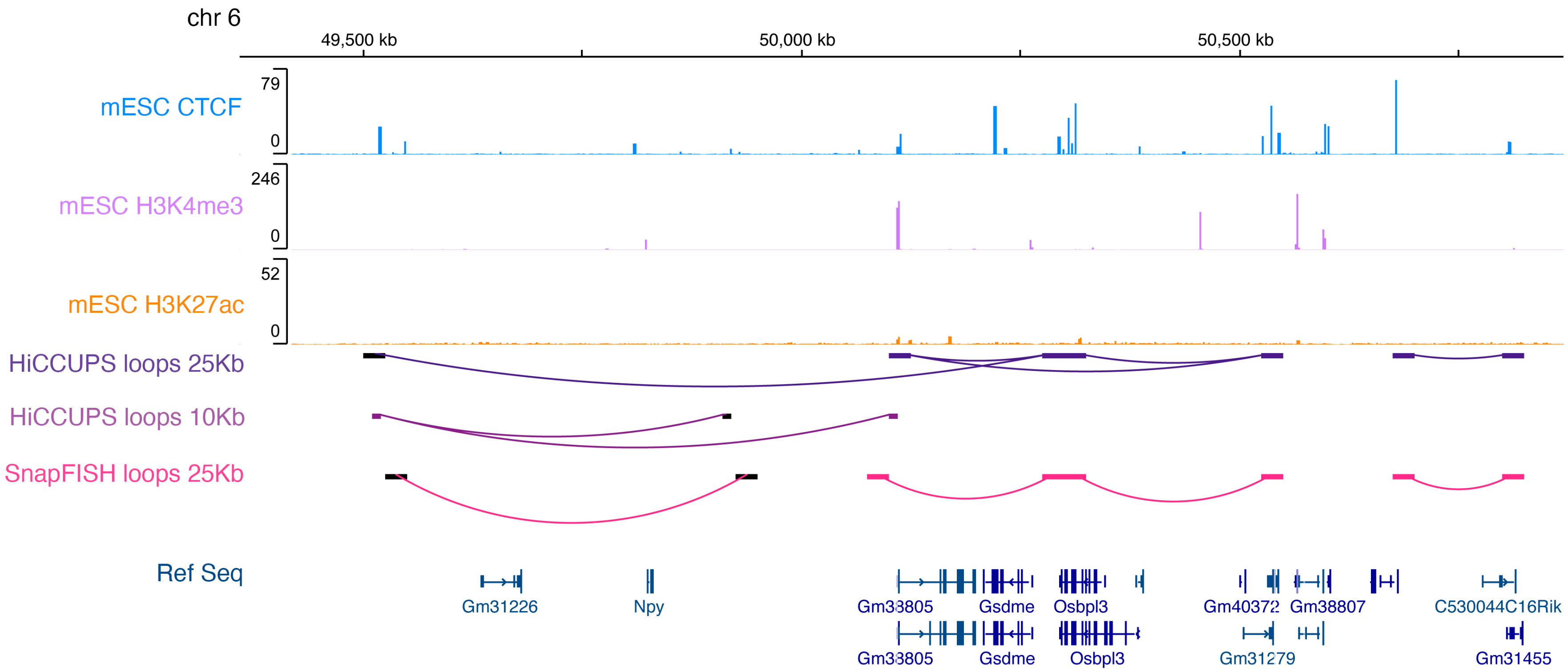

C. One SnapFISH-identified loop in chromosome 14.

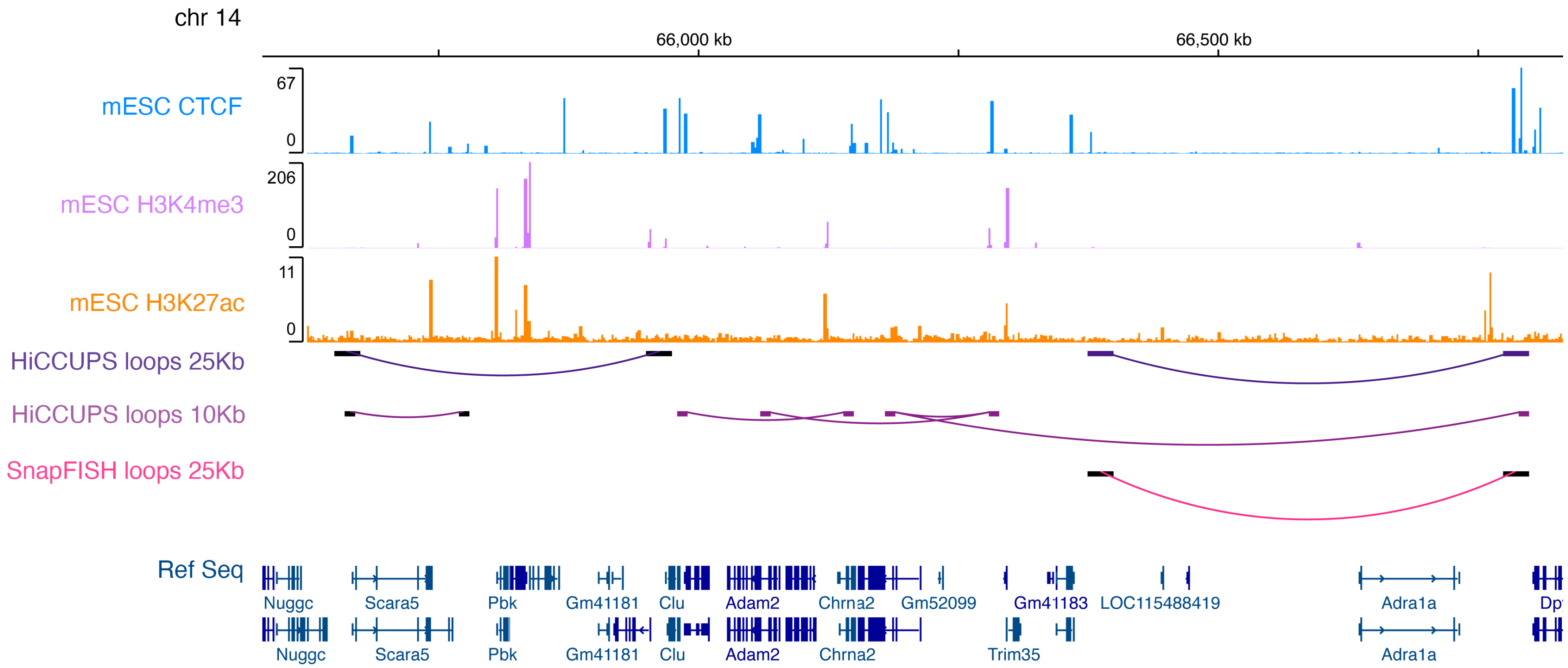

D. Four SnapFISH-identified loops in chromosome 16.

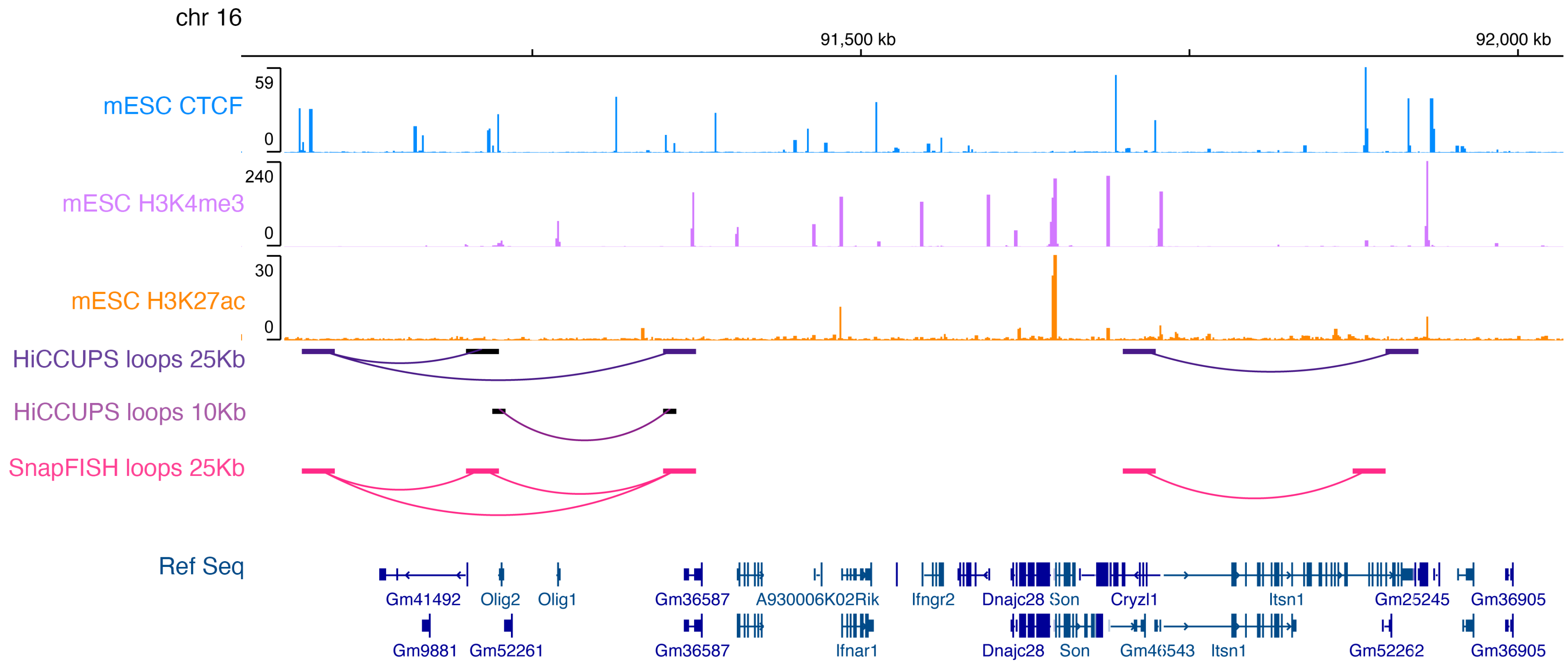

Figure S4. Applying SnapFISH to multiplexed DNA FISH data in mESCs. **A.** Average Euclidean distance (unit: nm) for all 5Kb resolution loci pairs calculated based on 1,416 CAST alleles (lower left panel) and 1,416 129 alleles (top right panel). Note that in 129 alleles, position #26 (chr3:34,725,000-34,730,000) is missing due to experimental error. **B.** Population-level contact frequency (i.e., the proportion of alleles with Euclidean distance smaller than the average Euclidean distance between two loci with 25Kb 1D genomic distance) for all 5Kb resolution loci pairs calculated based on 1,416 CAST alleles (lower left panel) and 1,416 129 alleles (top right panel). Similar to A, in 129 alleles, position #26 (chr3:34,725,000-34,730,000) is missing due to experimental error. **C.** 5Kb bin resolution log2 KR-normed Hi-C contact frequency matrix of mESC bulk Hi-C data (chr3:34.6Mb-34.805Mb) in the CAST allele (lower left panel) and the 129 allele (top right panel). **D.** Lower left panel: 44 loop candidates identified from 1,416 CAST alleles. Top right panel: 61 loop candidates identified from 1,416 129 alleles. The color is proportional to –log10 FDR at each loop candidate. Source data are provided.

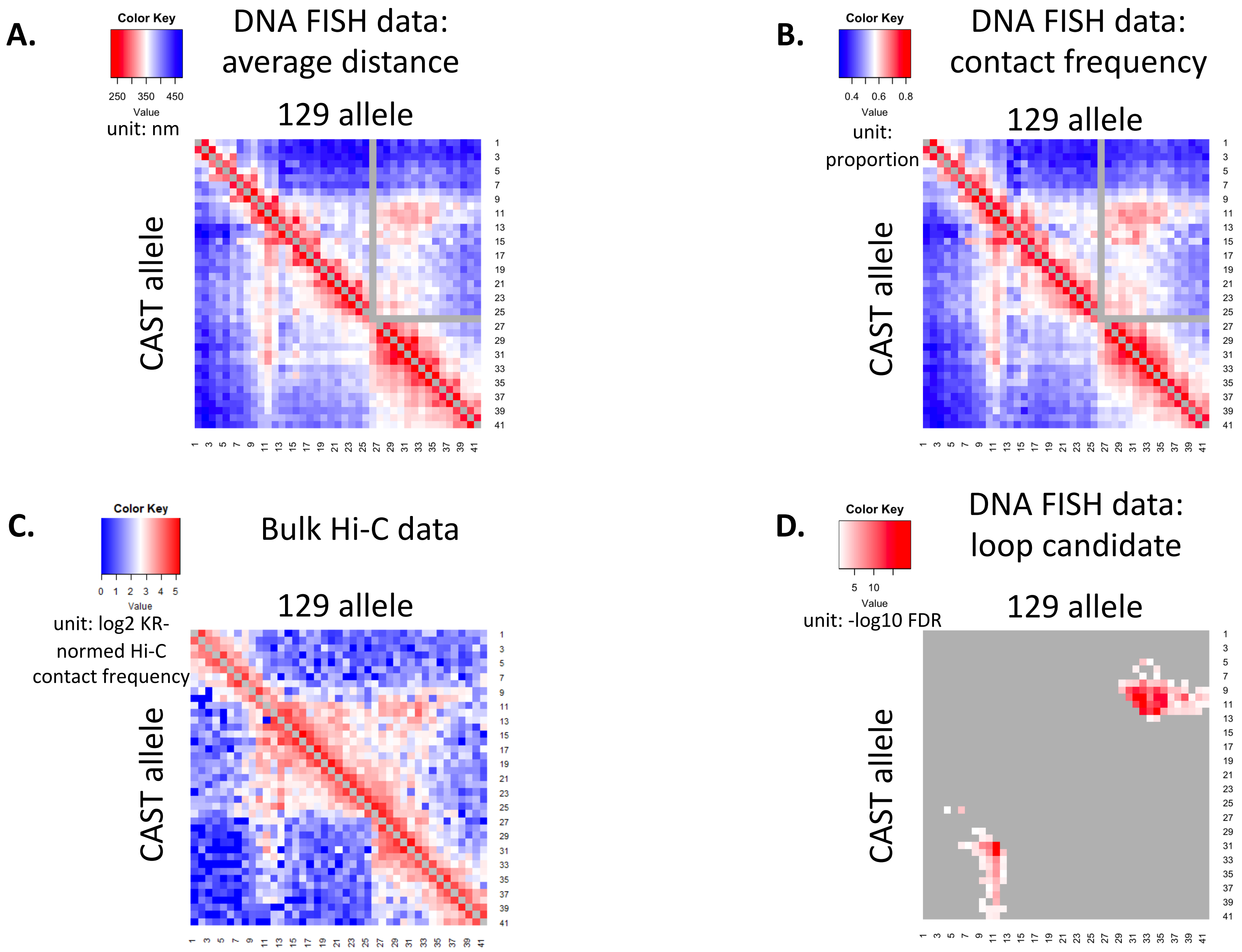
A.
Color Key
250 350 450
Value
unit: nm
DNA FISH data: average distance
129 allele
CAST allele
B.
Color Key
0.4 0.6 0.8
Value
unit: proportion
DNA FISH data: contact frequency
129 allele
CAST allele
C.
Color Key
0 1 2 3 4 5
Value
unit: log2 KR-normed Hi-C contact frequency
Bulk Hi-C data
129 allele
CAST allele
D.
Color Key
5 10
Value
unit: -log10 FDR
DNA FISH data: loop candidate
129 allele
CAST allele
1 3 5 7 9 11 13 15 17 19 21 23 25 27 29 31 33 35 37 39 41

Figure S5. Benchmark the sensitivity of SnapFISH with different numbers of alleles and alleles with different levels of targeted segment detection efficiency. A. SnapFISH identified the *Sox2* enhancer-promoter loop with more than 200 CAST alleles. **B.** SnapFISH identified the *Sox2* enhancer-promoter loop with more than 100 129 alleles. **C.** SnapFISH identified the *Sox2* enhancer-promoter loop with 472 129 alleles with all three different levels of targeted segment detection efficiency. SnapFISH identified *Sox2* enhancer-promoter loop with 472 CAST alleles with high and median levels of targeted segment detection efficiency. SnapFISH reported 3 false positives with 472 CAST alleles with a low level of targeted segment detection efficiency. Source data are provided.

A. SnapFISH identified *Sox2* enhancer-promoter loop with more than 200 CAST alleles.

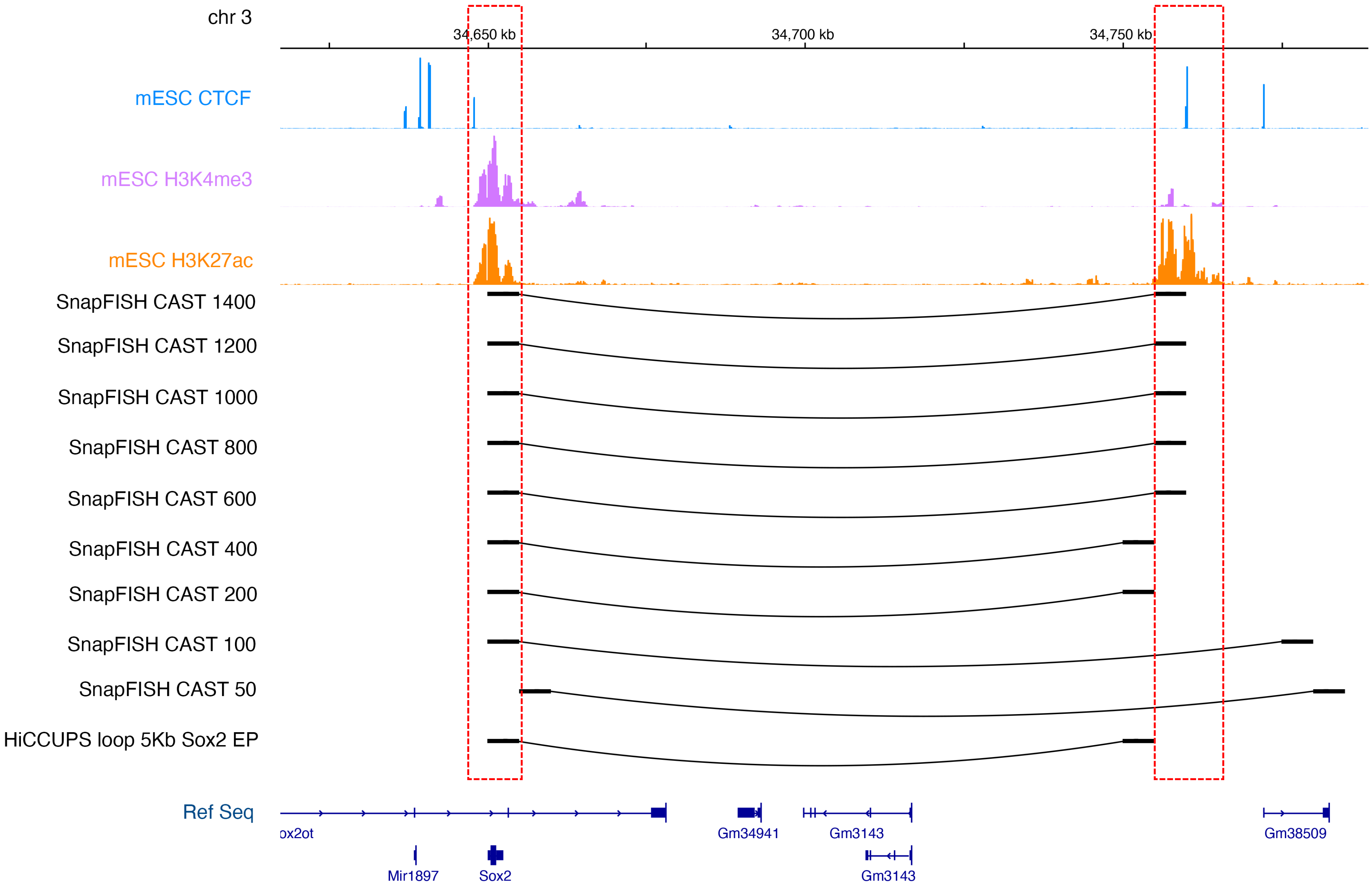

B. SnapFISH identified *Sox2* enhancer-promoter loop with more than 100 129 alleles.

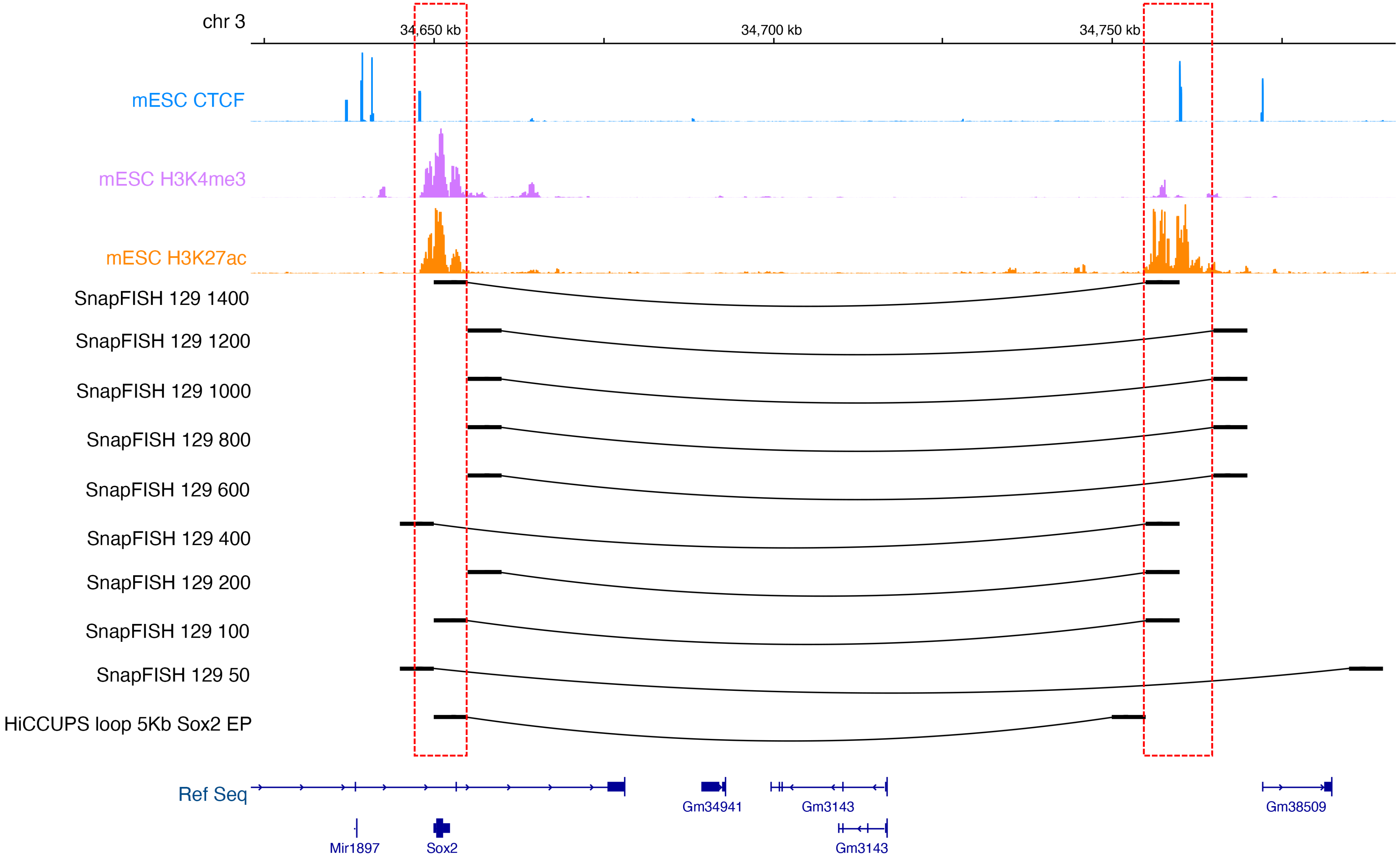

C. SnapFISH identified *Sox2* enhancer-promoter loop with 472 CAST alleles or 472 129 alleles with three different levels of targeted segment detection efficiency.

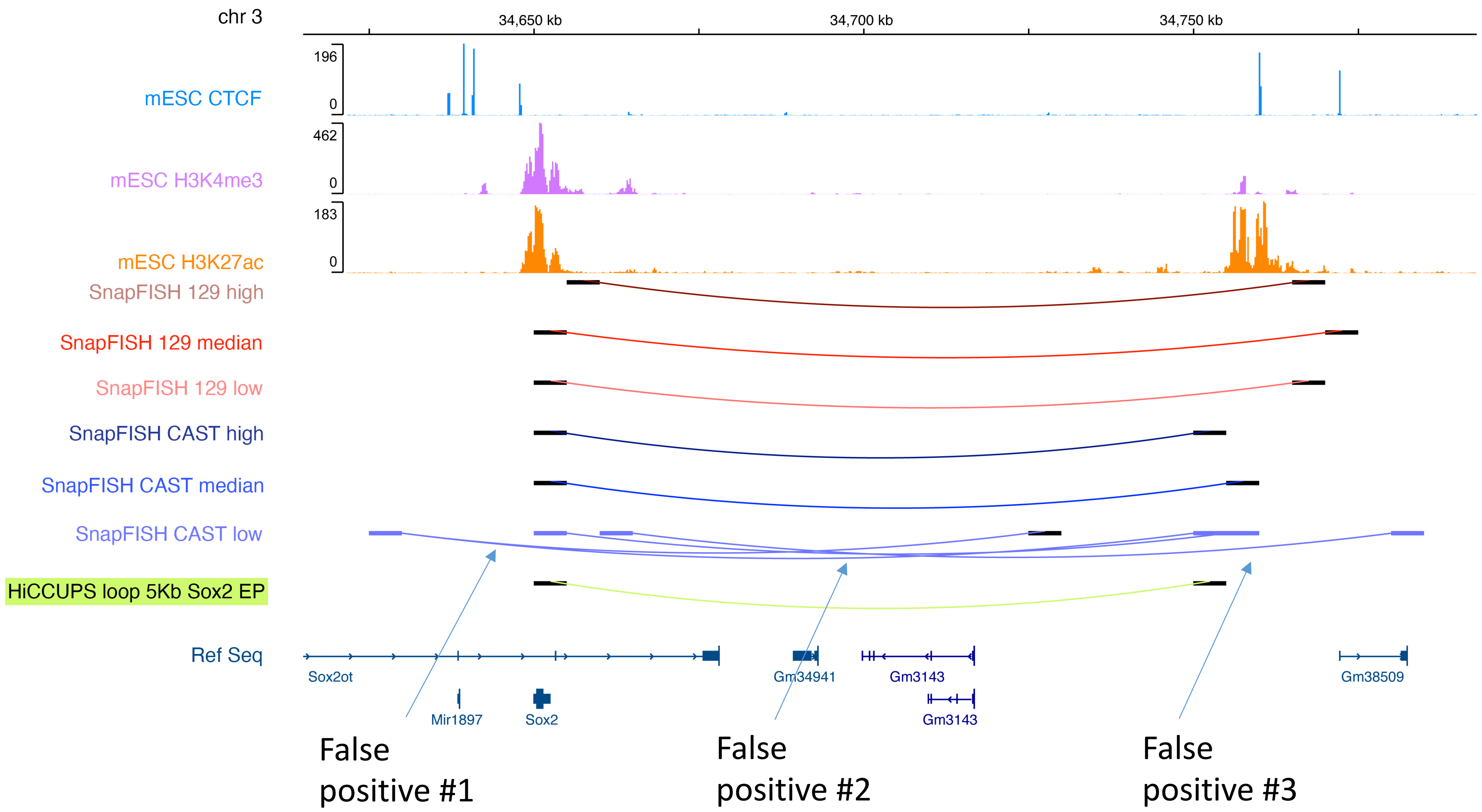

Figure S6. SnapFISH identified an enhancer-promoter loop in chromosome 14 from DNA seqFISH+ data in mouse astrocytes (Astro). The top five tracks are ATAC-seq, H3K4me1, H3K4me3, H3K27ac and RNA-seq data from astrocytes (Astro for short). The bottom two tracks are SnapFISH-identified 25Kb bin resolution loops and the Refseq gene annotation. The left anchor (dashed red box on the left) overlaps an ATAC peak. The right anchor (dashed red box on the right) is at the promoter of gene Adra1a, which is near strong H3K4me3, H3K4me1 and H3K27ac peaks. Source data are provided.

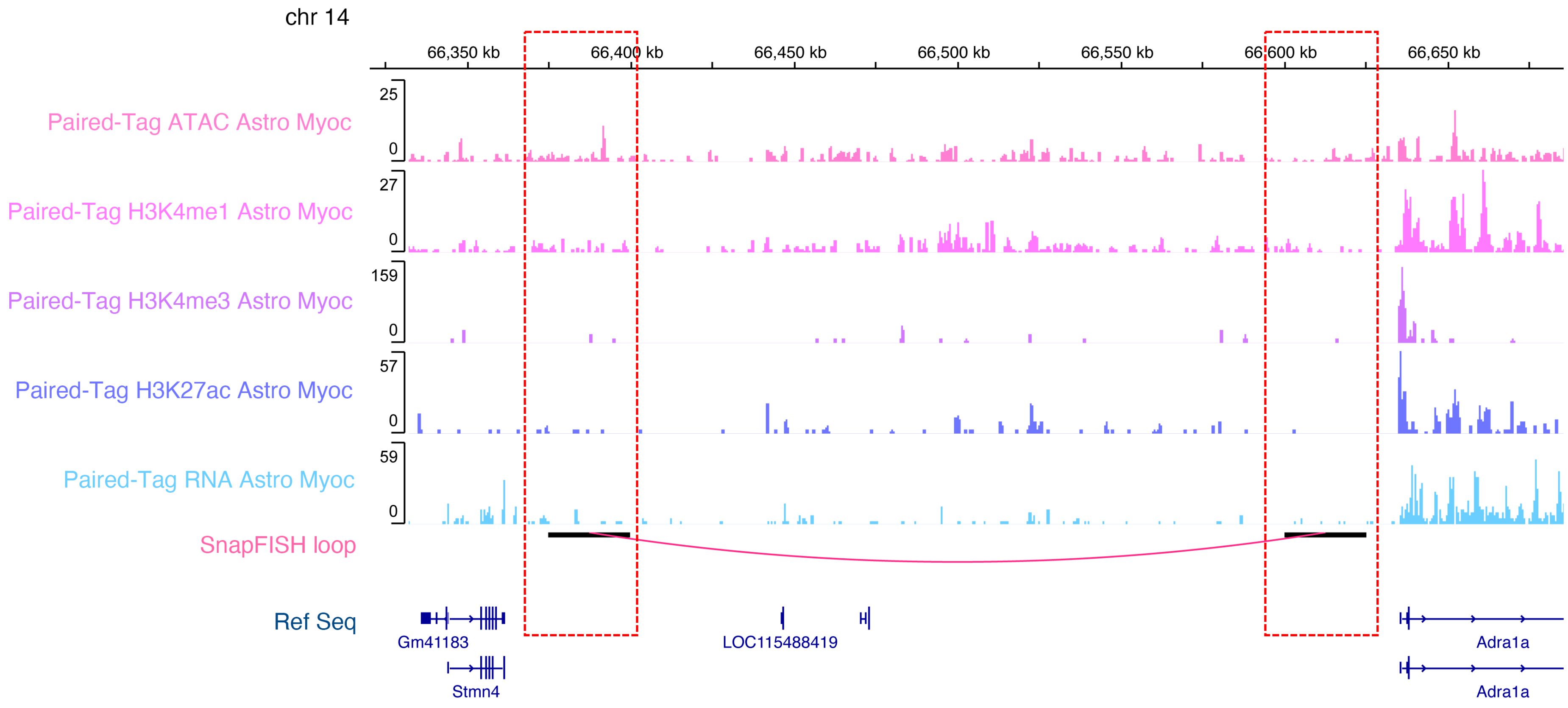

Figure S7. The reproducibility of SnapFISH-identified loops between biological replicates. A. SnapFISH identified loops in chromosome 16 from two biological replicates of mESC DNA seqFISH+ data. The top three tracks are mESC CTCF, H3K4me3 and H3K27ac ChIP-seq data. The middle four tracks are HiCCUPS-identified loops from mESC bulk Hi-C data at 10Kb bin and 25Kb bin resolution, SnapFISH-identified loops from replicate #1 (Rep1) and replicate #2 (Rep2), respectively. The bottom track is the Refseq gene annotation. **B.** SnapFISH identified loops in chromosome 16 from three biological replicates of DNA seqFISH+ data in mouse excitatory neurons. The top five tracks are ATAC-seq, H3K4me1, H3K4me3, H3K27ac and RNA-seq data from cortical excitatory neurons L2/3 collected from mouse frontal cortex tissue (FC_L2_3 for short). The middle three tracks are SnapFISH-identified loops in replicate #1 (Rep1), replicate #2 (Rep2) and replicate #3 (Rep3), respectively. All loops are at 25Kb bin resolution. The bottom track is the Refseq gene annotation. Source data are provided.

A. SnapFISH identified loops in chromosome 16 from two biological replicates of mESC DNA seqFISH+ data.

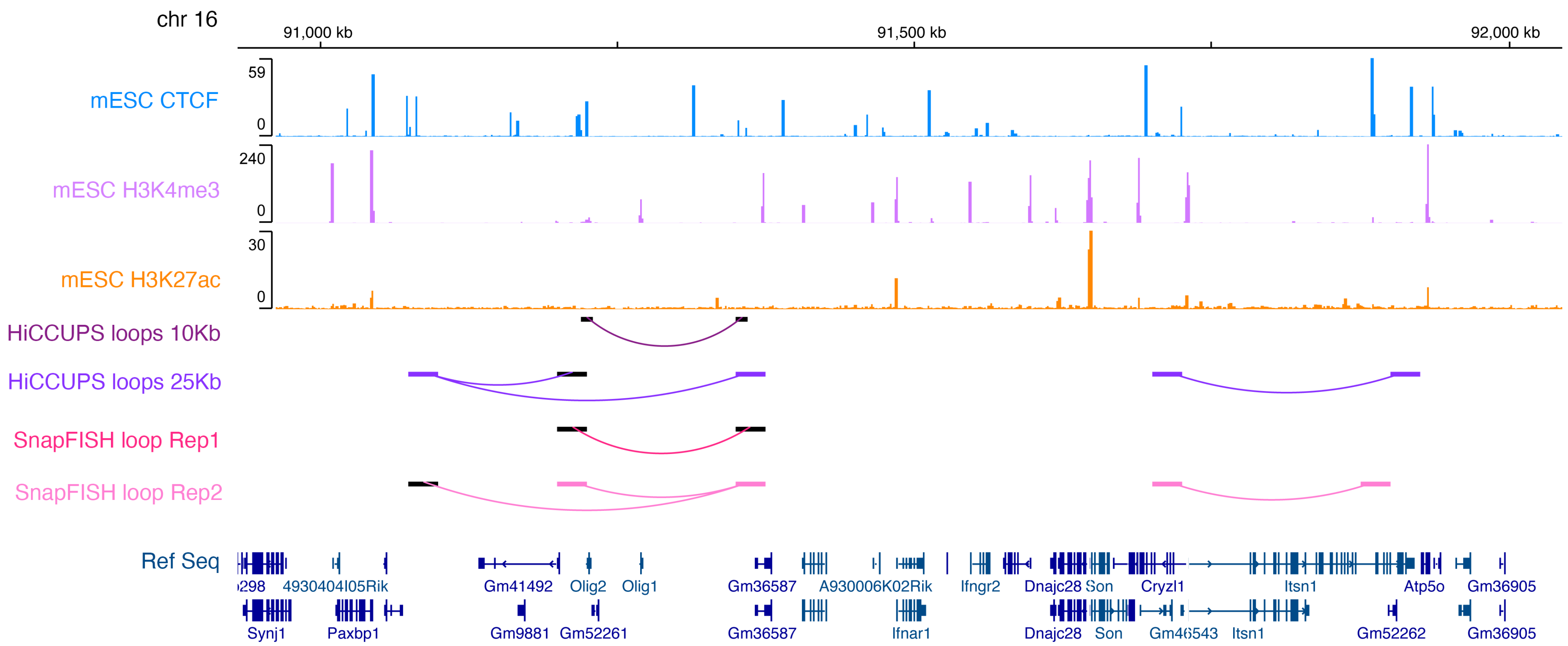

B. SnapFISH identified loops in chromosome 16 from three biological replicates of DNA seqFISH+ data in mouse excitatory neurons.

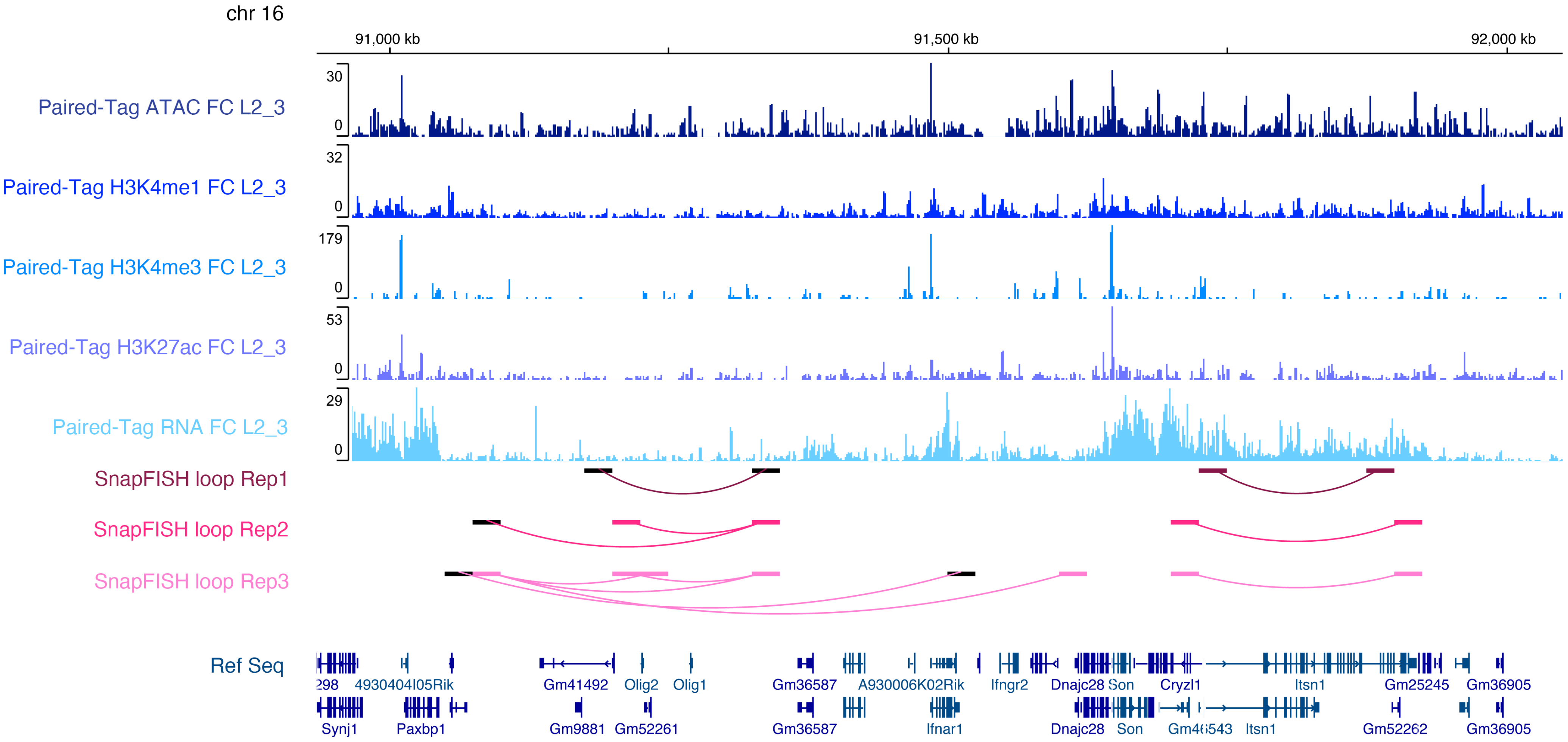

Figure S8. SnapFISH is robust to measurement errors in multiplexed DNA FISH data. A. mESC ORCA data. The top three tracks are mESC CTCF, H3K4me3 and H3K27ac ChIP-seq data. The middle three tracks are SnapFISH-identified loops from mESC ORCA data with low, median and high levels of measurement error. The bottom track is the Refseq gene annotation. **B.** mESC chromatin tracing data in the 129 alleles. The top three tracks are mESC CTCF, H3K4me3 and H3K27ac ChIP-seq data. The middle three tracks are SnapFISH-identified loops from mESC chromatin tracing data with low, median and high levels of measurement error. The bottom track is the Refseq gene annotation. Source data are provided.

A. OCRA data from mESCs.

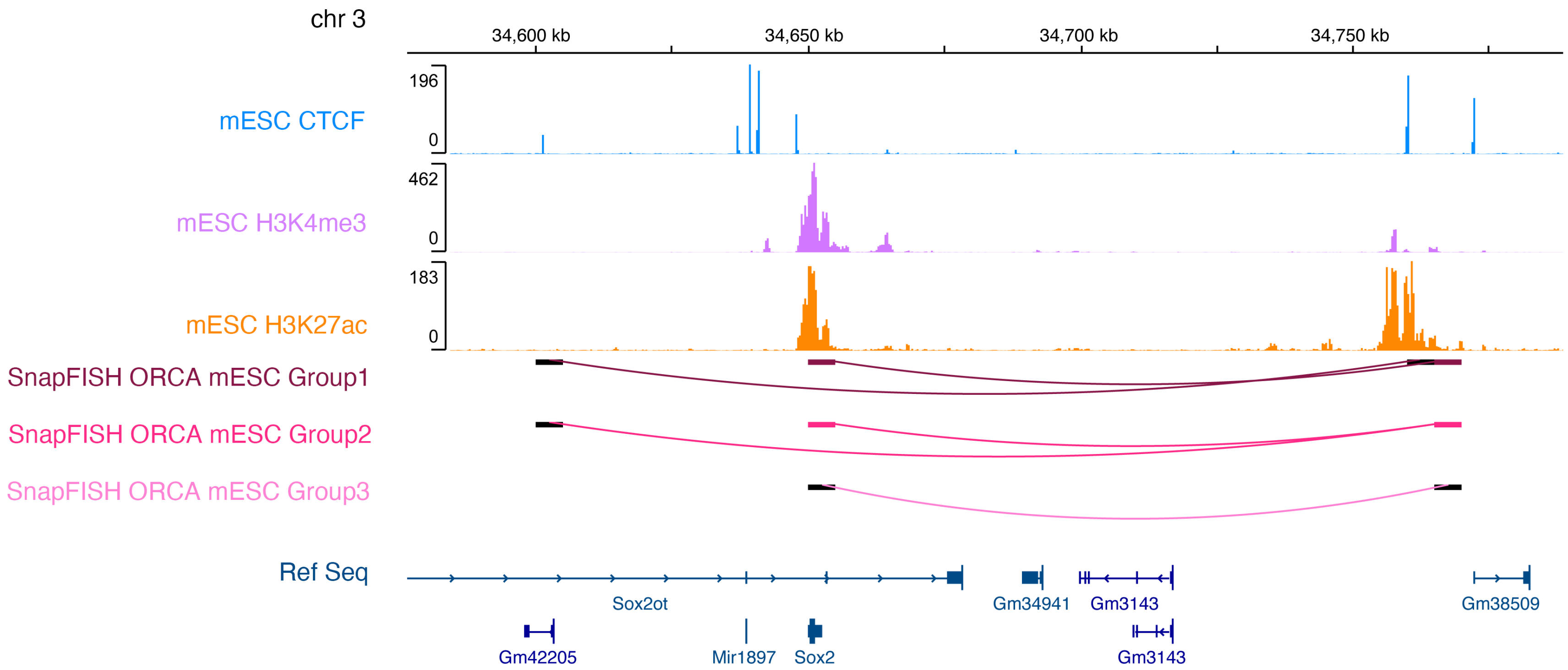

B. mESC chromatin tracing data in the 129 alleles.

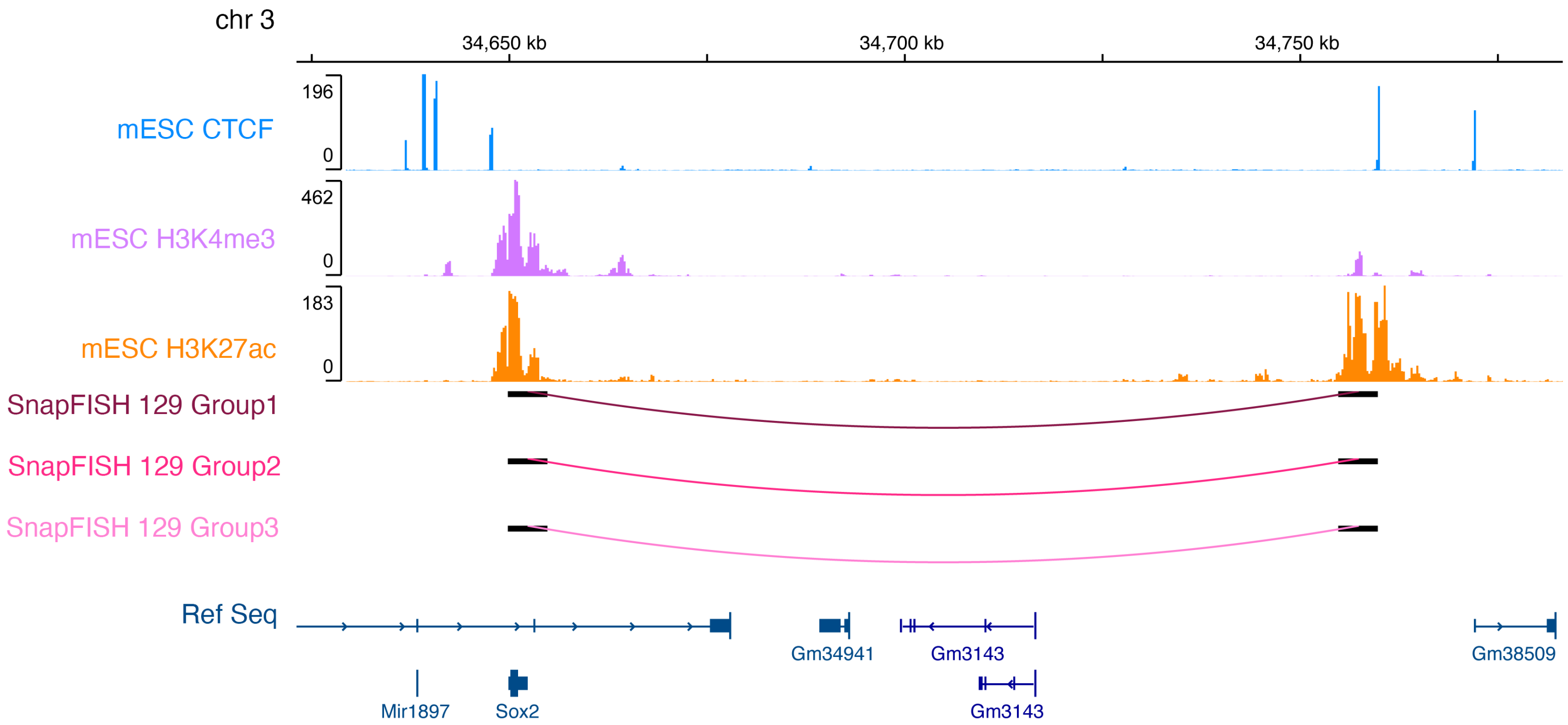

Figure S9. Distribution of Euclidean distance at the *Sox2* enhancer-promoter loop in the CAST allele (A), and the 129 allele (B). Source data are provided.

A.

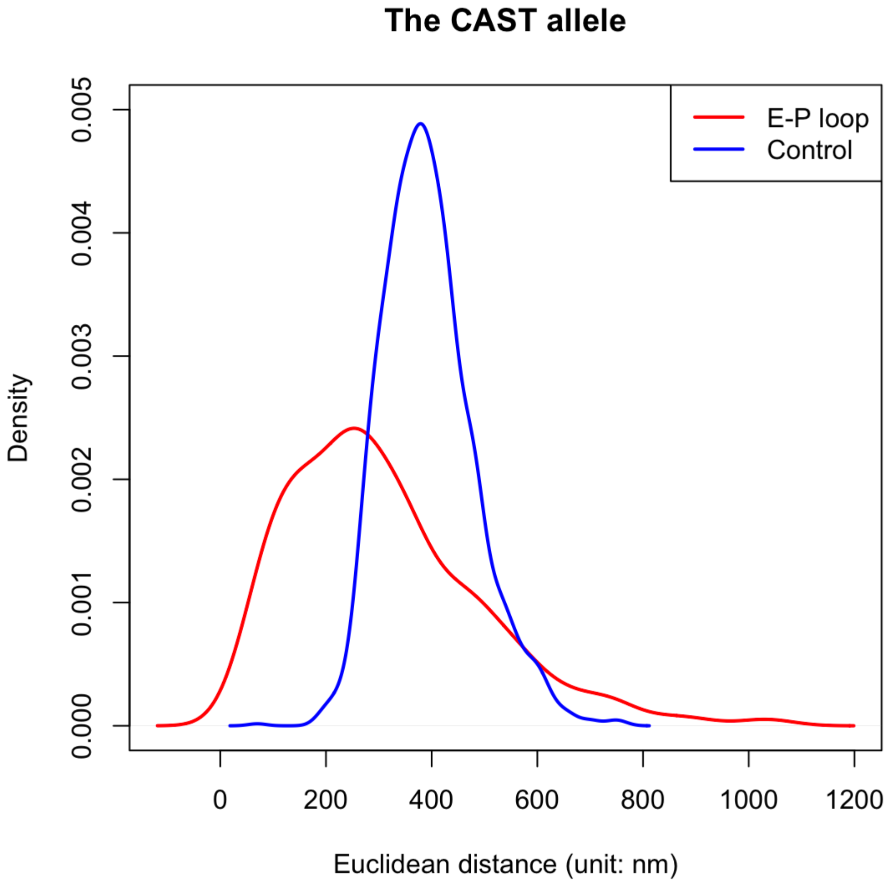

B.

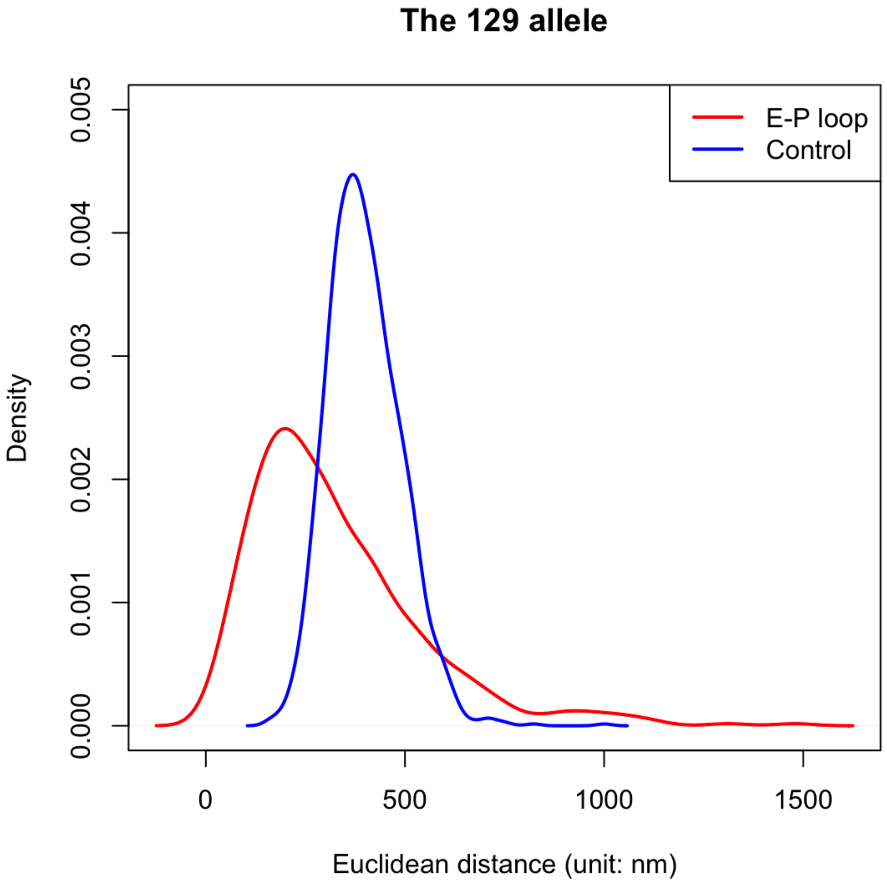

Figure S10. Scatter plot of the Euclidean distance at the *Sox2* enhancer-promoter loop against the average Euclidean distance in its local neighborhood control regions in the CAST allele (A), and the 129 allele (B).
Source data are provided.

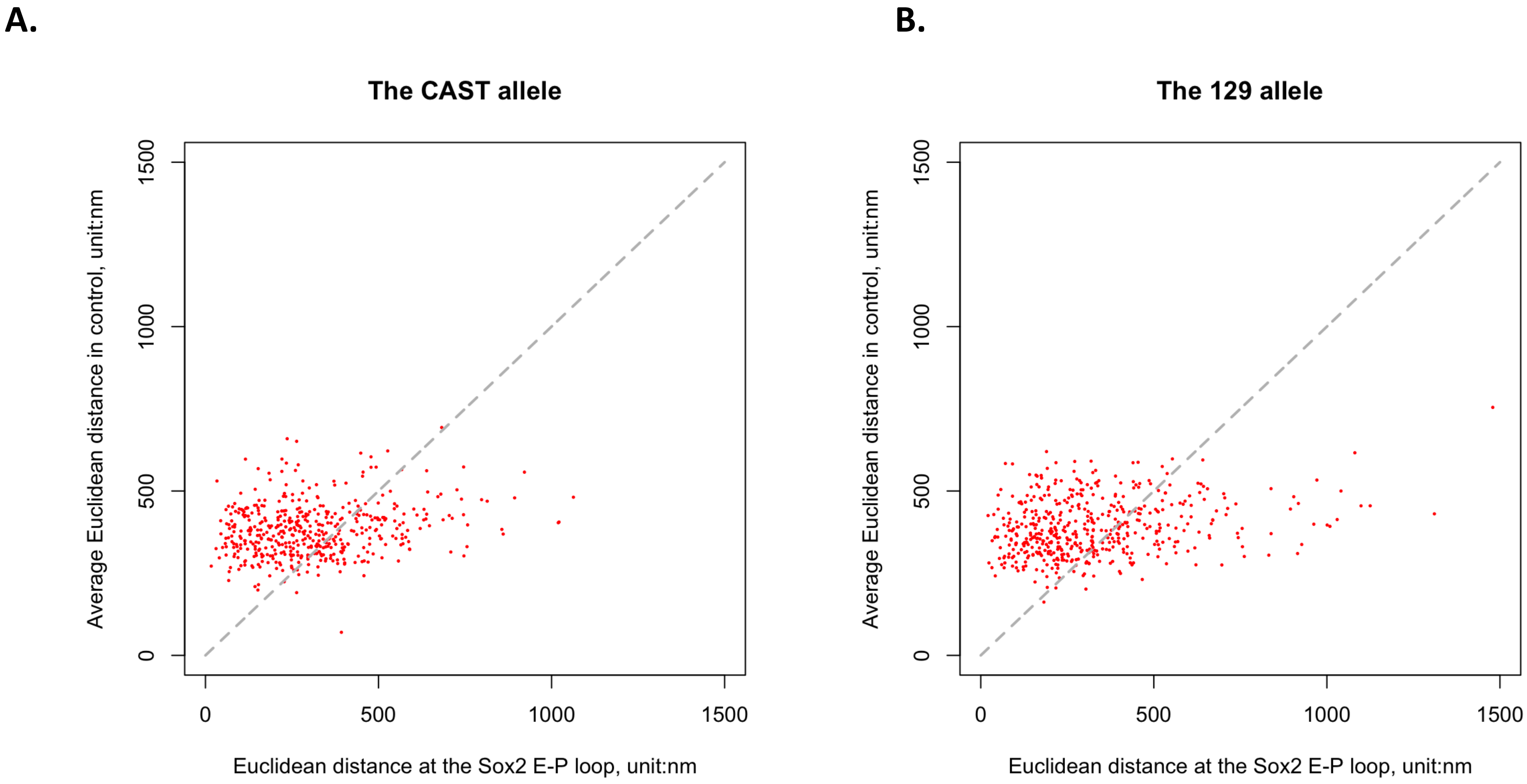

Figure S11. SnapFISH running time with different numbers of alleles. Source data are provided.

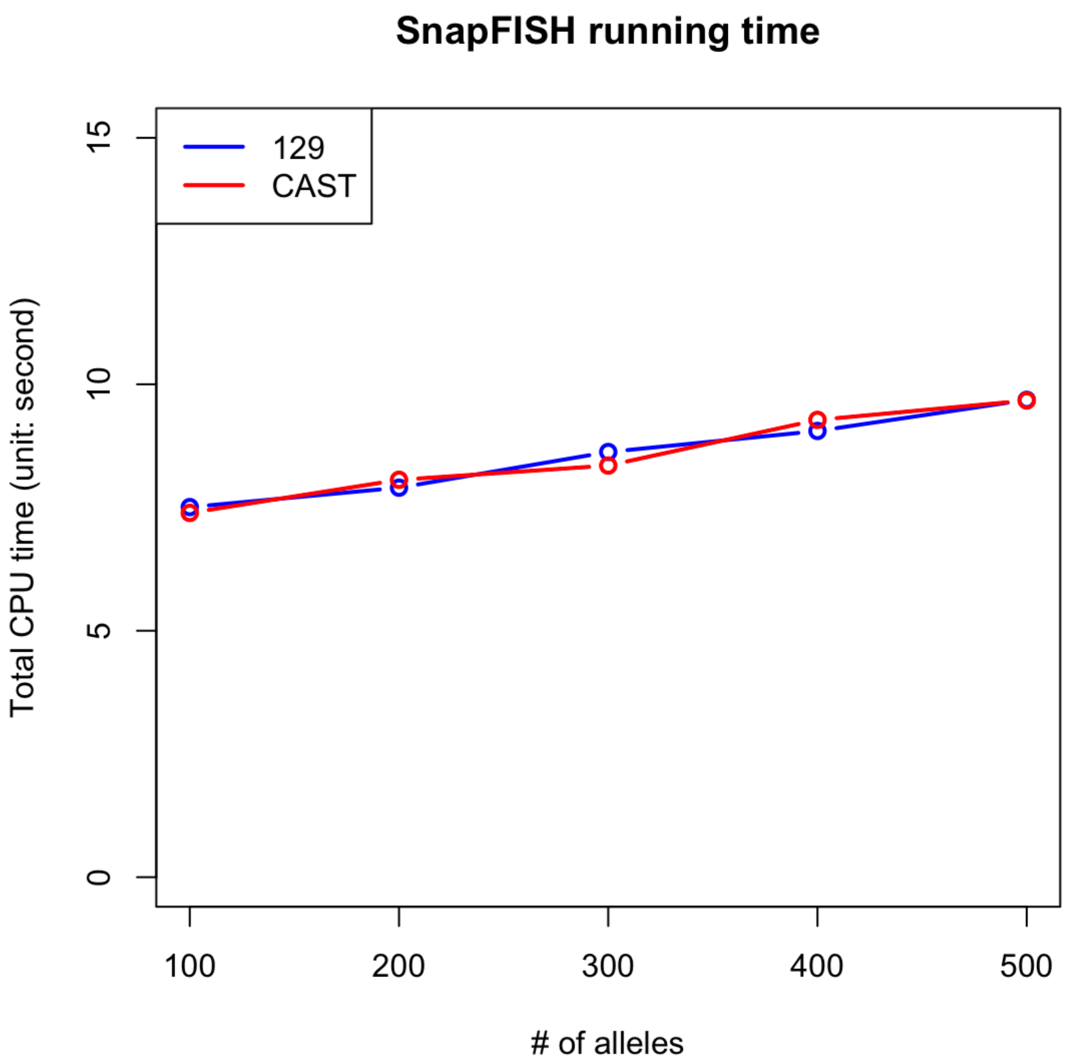

Supplement: Supplementary file 1 — Supplementary Information [file 41467_2023_40658_MOESM1_ESM.pdf]
