# Supplementary material for: SnapFISH: a computational pipeline to identify chromatin loops from multiplexed DNA FISH data
Source: Nat Commun. 2023 Aug 12;14:4873. doi: 10.1038/s41467-023-40658-3 (PMC10423204; doi:10.1038/s41467-023-40658-3)
Supplement: Supplementary file 3 — Description of Additional Supplementary Files [file 41467_2023_40658_MOESM3_ESM.pdf]

Description of Additional Supplementary Files:

Supplementary Dataset 1: ORCA data original used in the Mateo et al study (PMID: 30886393), file: Sox2_B1_T1_core.csv, 10.4Mb

Supplementary Dataset 2: Supplementary Tables
