# Supplementary material for: SnapFISH: a computational pipeline to identify chromatin loops from multiplexed DNA FISH data
Source: Nat Commun. 2023 Aug 12;14:4873. doi: 10.1038/s41467-023-40658-3 (PMC10423204; doi:10.1038/s41467-023-40658-3)
Supplement: Supplementary file 2 — Peer Review File [file 41467_2023_40658_MOESM2_ESM.pdf]

nature portfolio

Peer Review File

SnapFISH: a computational pipeline to identify chromatin loops from multiplexed DNA FISH data

Open Access This file is licensed under a Creative Commons Attribution 4.0 International License, which permits use, sharing, adaptation, distribution and reproduction in any medium or format, as long as you give appropriate credit to the original author(s) and the source, provide a link to the Creative Commons license, and indicate if changes were made. In the cases where the authors are anonymous, such as is the case for the reports of anonymous peer reviewers, author attribution should be to 'Anonymous Referee' followed by a clear attribution to the source work. The images or other third party material in this file are included in the article's Creative Commons license, unless indicated otherwise in a credit line to the material. If material is not included in the article's Creative Commons license and your intended use is not permitted by statutory regulation or exceeds the permitted use, you will need to obtain permission directly from the copyright holder. To view a copy of this license, visit http://creativecommons.org/licenses/by/4.0/.

Editorial Note: This manuscript has been previously reviewed at another journal that is not operating a transparent peer review scheme. This document only contains reviewer comments and rebuttal letters for versions considered at *Nature Communications*.

Reviewer #1 (Remarks to the Author):

The authors have adequately addressed my previous concerns and in my opinion strengthened the manuscript, particularly with the inclusion of the additional analyses relating to the statistical framework used and the effect of localization uncertainty. The posting of the software to PyPI and the changes to the GitHub repo also strengthen the work. I support publication in Nature Communications.

Reviewer #2 (Remarks to the Author):

The authors addressed all my previous comments (reviewer#2), which I wrote for their previous Nature Methods submission.
The paper has further improved and is an important contribution to field. It should be published in Nature Communications.

Reviewer #3 (Remarks to the Author):

The authors have addressed all my comments and suggestions. I support the acceptance of this revised manuscript as is.
